# Upgrading the Topical Delivery of Poorly Soluble Drugs Using Ionic Liquids as a Versatile Tool

**DOI:** 10.3390/ijms22094338

**Published:** 2021-04-21

**Authors:** Rita Caparica, Ana Júlio, Filipe Fernandes, Maria Eduarda M. Araújo, João Guilherme Costa, Tânia Santos de Almeida

**Affiliations:** 1 CBIOS—Universidade Lusófona’s Research Center for Biosciences & Health Technologies, Campo Grande 376, 1749-024 Lisboa, Portugal; a21707353@alunos.ulht.pt (R.C.); ana.julio@ulusofona.pt (A.J.); 2 Department of Biomedical Sciences, University of Alcalá, Ctra. Madrid-Barcelona Km. 33.600, Alcalá de Henares, 28871 Madrid, Spain; 3 School of Sciences and Health Technologies, Lusófona University, Campo Grande 376, 1749-024 Lisboa, Portugal; a21603423@alunos.ulht.pt; 4 CQE, and Department of Chemistry and Biochemistry, Faculty of Sciences, University of Lisbon, Campo Grande, 1749-016 Lisboa, Portugal; mearaujo@fc.ul.pt

**Keywords:** ionic liquids, HaCaT, phenolic compounds, solubility studies, upgraded O/W emulsions, stability studies

## Abstract

Numerous studies are continuously being carried out in pursuit of formulations with higher performance. Problems such as poor drug solubility, which hinders drug incorporation into delivery systems and bioavailability, or limitations concerning the stability and performance of the formulations may cause difficulties, since solving all these drawbacks at once is a huge challenge. Ionic liquids (ILs), due to their tunable nature, may hypothetically be synthesized for a particular application. Therefore, predicting the impact of a particular combination of ions within an IL in drug delivery could be a useful strategy. Eight ILs, two choline amino acid ILs, two imidazole halogenated ILs, and four imidazole amino acid ILs, were prepared. Their applicability at non-toxic concentrations, for improving solubility and the incorporation of the poorly soluble, ferulic, caffeic, and *p*-coumaric acids, as well as rutin, into topical emulsions, was assessed. Next, the impact of the ILs on the performance of the formulations was investigated. Our study showed that choosing the appropriate IL leads to a clear upgrade of a topical emulsion, by optimizing multiple features of its performance, such as improving the delivery of poorly soluble drugs, altering the viscosity, which may lead to better sensorial features, and increasing the stability over time.

## 1. Introduction

Ionic liquids (ILs) have emerged as valuable tools to solve a wide range of challenges in the pharmaceutical field. Consequently, the number of studies that have considered their applicability in this area has increased considerably [1,2,3]. ILs are usually defined as organic salts, which are liquid at temperatures below 100 °C, or in some cases liquid at room temperature (RTILs) [4,5,6,7]. These materials are structurally composed of an organic cation (e.g., ammonium, imidazolium, pyridinium, cholinium) and an inorganic (e.g., halides, sulfates) or organic (e.g., phosphates, acetate) anion [8,9,10]. They have several unique and valuable physicochemical properties, such as low volatility and vapor pressure [4,5,6,8], non-flammability [4], high ionic conductivity, thermal and chemical stability [10,11], and the possibility of being recycled [5]. Furthermore, they present good a capability for dissolving inorganic, organic, and polymeric substances [4,6,12], which is very relevant when it is intended to use these salts as functional excipients in delivery systems. Furthermore, the high susceptibility of ILs to structural modifications allows their properties to be tailored according to a particular application, and this may be used to further reinforce their utility in different areas [4,5,6]. Thus, different combinations of the cations and anions that constitute them, and/or the attached substituents, may lead to specific physicochemical and biological properties, including hydrophobicity, impact on solubility, and toxicity (cyto- and ecotoxicity), among others [10,11,13]. Hence, when considering the applicability of ILs towards the development of improved drug delivery systems, the strategic tailoring of these materials may be crucial to ensuring their functionality, at non-toxic concentrations. To achieve this, cytotoxicity studies should always be considered beforehand.

**Figure 1:**
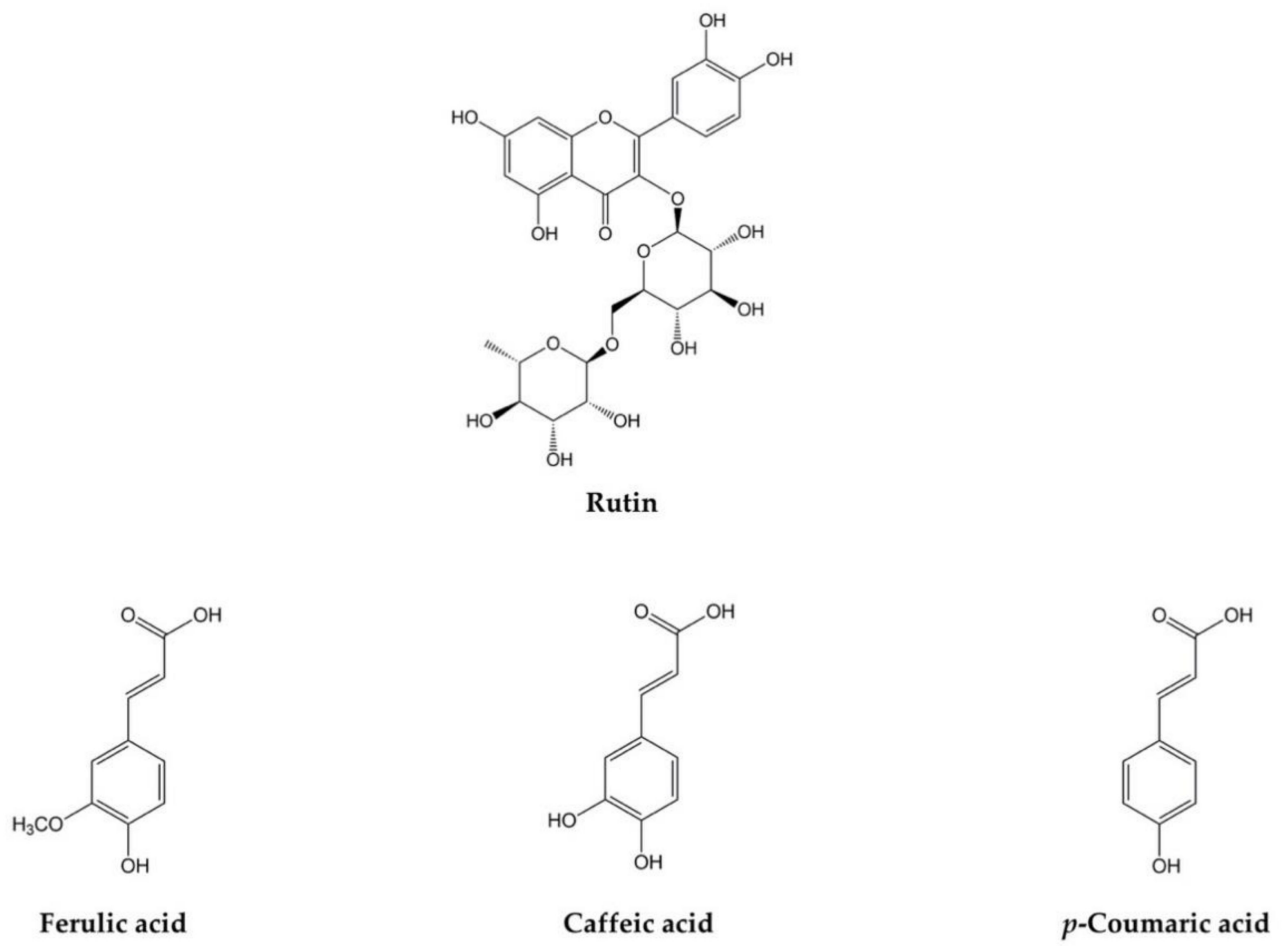
Chemical structures of the four studied phenolic compounds.

The pharmaceutical industry continues to face a series of challenges related to the discovery of innovative and effective drugs [4,5,6]. In fact, despite the promising pharmacological properties of many compounds, several have a very low solubility in water and, in some cases, in pharmaceutically accepted organic solvents. This then impairs, not only their bioavailability, but also their incorporation into drug delivery systems [6,9], which may impact the stability of the developed systems and hinder the delivery of the drugs. In this context, many studies have highlighted the pharmacological and cosmetic potential of some natural compounds [14,15]. Their diversity and unique properties have made them important tools for the research and development of new therapeutic agents [16,17]. Amongst these, phenolic compounds have attracted considerable interest for their wide variety of biological properties, including their antioxidant, antimicrobial, anti-inflammatory, and anticancer activities [17,18,19,20], but many of them are poorly soluble in water. Rutin, as well as ferulic, caffeic, and *p*-coumaric acids (Figure 1), are examples of phenolic compounds of pharmaceutical and cosmetic interest [14,21,22], but with very low water solubility, which impairs their applicability.

In this setting, ILs have proven to be a useful approach to increase drug solubility [5,6,23] and drug loading [24,25,26,27,28] into drug delivery systems. Nonetheless, these abilities may considerably vary with the type of IL, particularly with their properties and structural composition. Additionally, the type of drugs studied is also relevant [6,9,23,29].

It has been shown that choline-amino acid ILs, can be better solubility promotors, compared with imidazole-based ILs, but the latter seem to be better suited to increase drug permeation through the skin [23], which could also be valuable. Hence, since both of these features may be valuable, particularly for topical delivery, combining the cations/anions of these classes of ILs may be a relevant strategy, and this approach will be studied herein. Moreover, it is also important to further explore other functionalities of these ILs in an attempt to use ILs that may convey multiple functionalities, and thus ensure their optimized applicability. Bearing these goals in mind, the influence of these ILs on the incorporation of poorly soluble drugs into oil-in-water (O/W) emulsions, and their effect on the preparation and stability of the developed formulations, will also be assessed. Therefore, it is important to strategically continue to evaluate the functionalities of ILs, in terms of their applicability in drug delivery, and even to explore which ILs may convey multiple functionalities, to ensure optimized applicability.

Consequently, herein, eight different ILs, combining an imidazolium or cholinium cation with bromide or amino acids anions, were prepared to evaluate the impact of the different combinations, of cation–anion, on their functionality in topical delivery. To achieve this, the impact of the ILs on the cell viability of human keratinocytes (HaCaT), as well as their effect on the solubility of the poorly water-soluble compounds ferulic, caffeic, and *p*-coumaric acids, and rutin, were studied. Since these phenolic compounds may be of interest for topical applicability, namely as antioxidants, anti-inflammatories, or as anti-wrinkle agents [14,21,30,31], and as their incorporation into topical delivery systems is impaired and could negatively impact the stability of the formulations, the possible use of ILs to improve the performance of O/W emulsions, was assessed.

## 2. Results and Discussion

To evaluate how different cation/anion combinations may lead to ILs with multiple functionalities, useful for the topical delivery of poorly soluble active compounds, different ILs were synthesized.

### 2.1. Synthesis of ILs

**Scheme 1:**
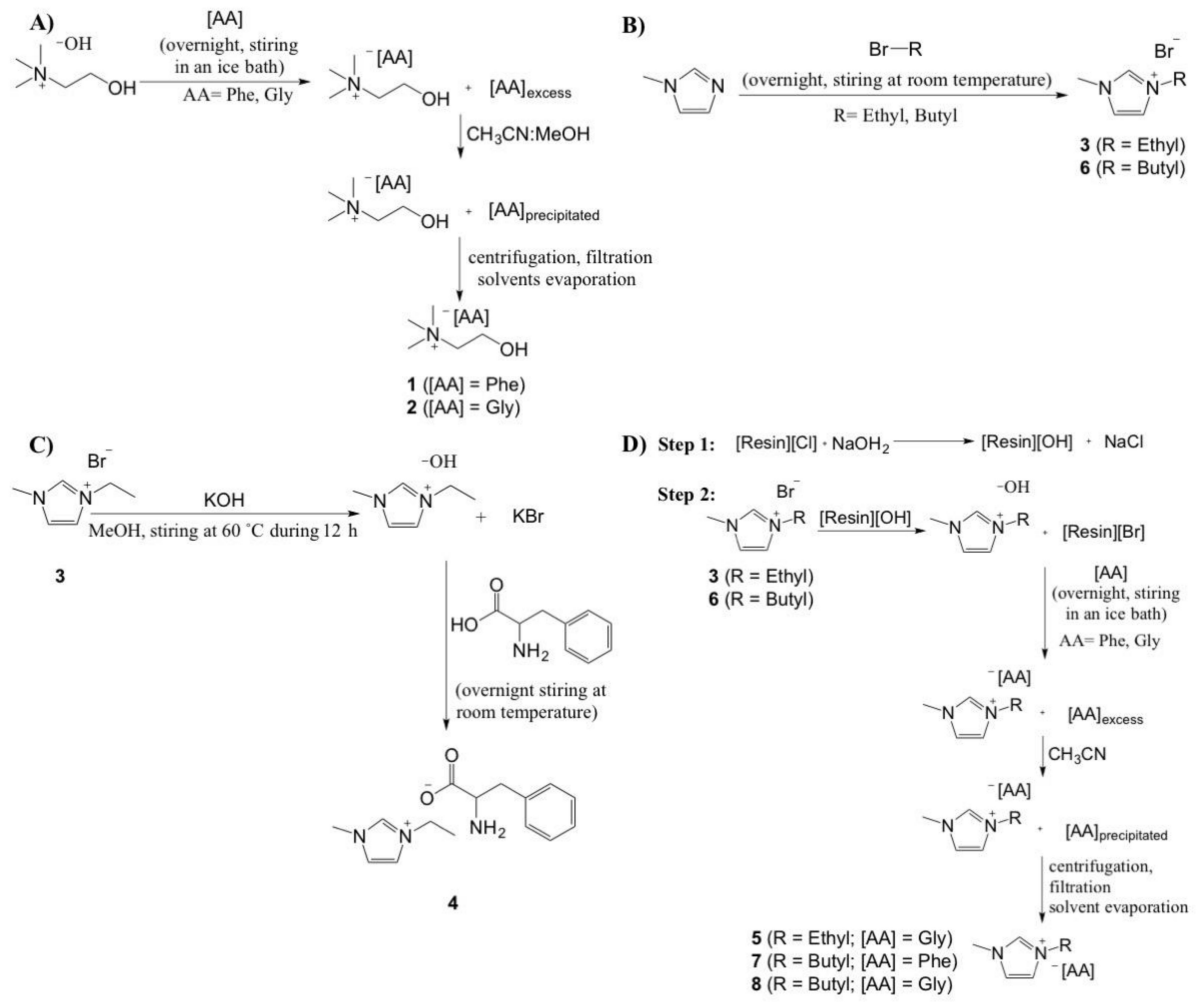
Synthetic procedures used to obtain: (**A**) the ILs [Cho][Phe] (**1**) and [Cho][Gly] (**2**); (**B**) the ILs [Emim][Br] (**1**) and [Bmim][Br] (**6**); (**C**) the IL [Emim][Phe] (**4**); and (**D**) the ILs [Emim][Gly] (**5**), [Bmim][Phe] (**7**), and [Bmim][Gly] (**8**).

Eight ILs were synthesized using different synthetic methods (Scheme 1) and combining different cations and anions. Since the choline-amino acid ILs have been considered less toxic, and useful as drug solubility promotors [4,6,23,32], two choline amino acid ILs were prepared, (2-hydroxyethyl)trimethylammonium phenylalaninate [Cho][Phe] (**1**) and (2-hydroxyethyl)trimethylammonium glycinate [Cho][Gly] (**2**). Then, two imidazole-halogenated based ILs, 1-ethyl-3-methylimidazolium bromide [Emim][Br] (**3**) and 1-butyl-3-methylimidazolium bromide [Bmim][Br] (**6**), were synthesized. Even though these latter ILs have been considered more toxic, and thus less suited for pharmaceutical applicability, they have also been described as useful for enhancing drug permeation (mostly due to the side chain in the imidazolium cation), and thus taking advantage of this property may be useful, particularly for topical delivery.

Hence, this justifies, not only our interest in further studying this class of ILs, but also in preparing ILs containing imidazolium cations and amino acid anions, to assess if this combination could be a profitable strategy for topical applications. Consequently, 1-ethyl-3-methylimidazolium phenylalaninate [Emim][Phe] (**4**), 1-ethyl-3-methylimidazolium glycinate [Emim][Gly] (**5**), 1-butyl-3-methylimidazolium phenylalaninate [Bmim][Phe] (**7**), and 1-butyl-3-methylimidazolium glycinate [Bmim][Gly] (**8**) were also prepared. The eight ILs were obtained using different procedures, and only the methodologies that led to better yields are described in Section 3.2.

All ILs were characterized by FTIR, ^1^H NMR, and ^13^C NMR spectroscopy, and the spectroscopic data were according to the literature [12,33,34,35] published for the same compounds. The physical–chemical characteristics of the ILs and the yields from each synthesis are presented in Table 1.

Although recent studies have shown several favorable applications of ILs, their use for pharmaceutical and cosmetic applications is still limited. The major reason for this is the high toxicity of some ILs. Thus, since the aim of this work is to assess the potential use of these salts to improve topical delivery, evaluating the toxicity of the prepared ILs is essential, particularly considering the impact of different combinations of cations and anions. Specifically, it is key to uncover, beforehand, the upper concentration of each IL that might be included in the developed delivery systems, to ensure safety; and only then can we further evaluate if these materials may truly be a valuable technological approach to obtain topical systems with higher performance.

### 2.2. Impact of ILs on HaCaT Cells Viability

To assess the upper safer concentration of the [Cho][Br] salt and the ILs to be used in the following experiments, HaCaT human keratinocytes were exposed to these compounds (0–1%; 24 h), and their impact on cell viability was evaluated by MTT assay. This cell line was chosen since topical applicability was foreseen. The MTT assay is based on the assumption that the number of viable cells depends linearly on their mitochondrial activity and, therefore, is an in vitro tool commonly used to evaluate the cytotoxic potential of compounds [14].

Thus, the impact of the [Cho][Br] salt, the choline amino acid ILs ([Cho][Phe], [Cho][Gly]), the imidazole halogenated ILs ([Emim][Br], [Bmim][Br]), and the four-imidazole amino acid ILs ([Emim][Phe], [Bmim][Phe], [Emim][Gly], and [Bmim][Gly]), on HaCaT cells viability was evaluated.

**Figure 2:**
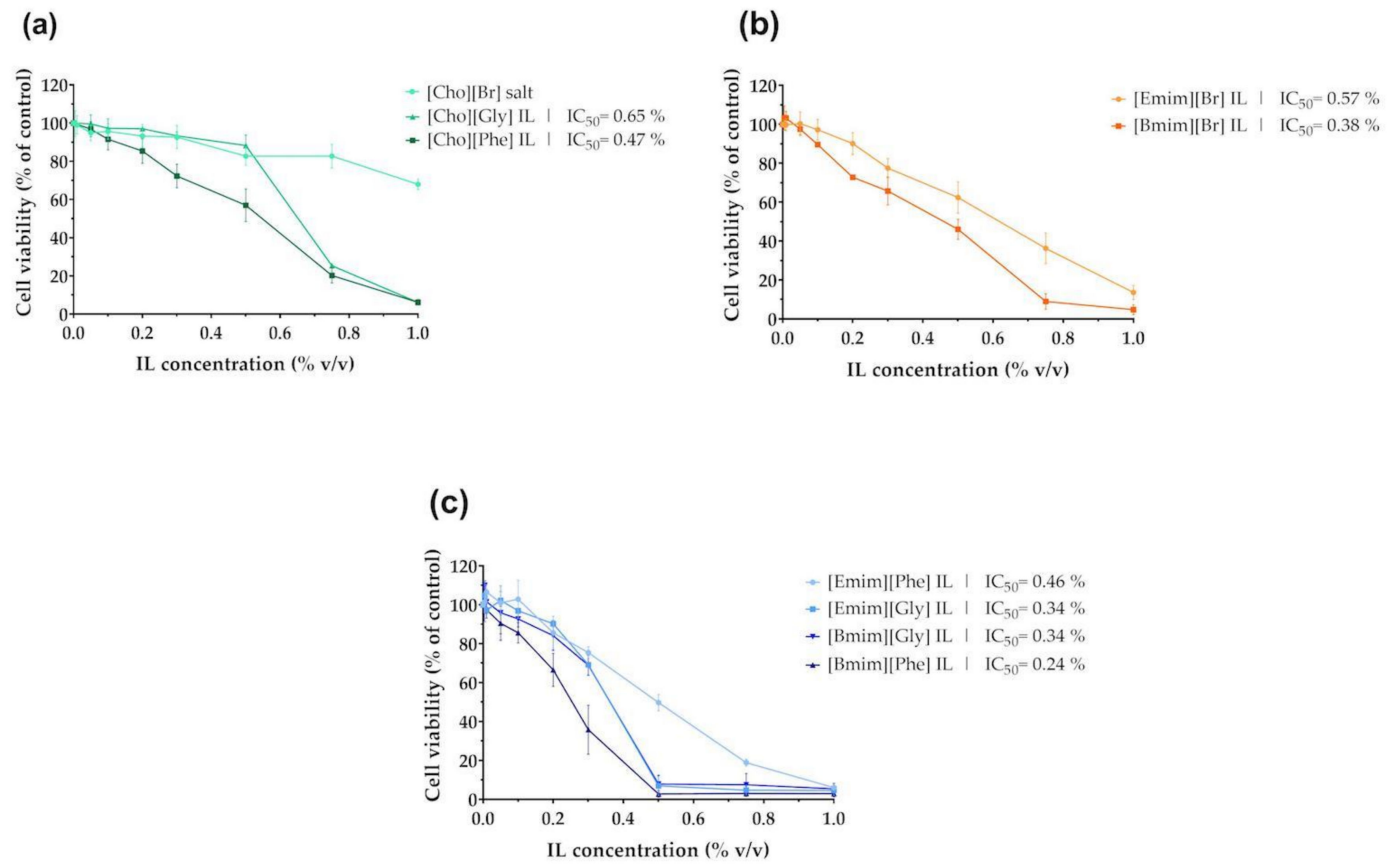
Cell viability of HaCaT cells exposed to choline amino acid-based ILs and [Cho][Br] salt (**a**), to imidazole-based ILs (**b**), and imidazole amino acid-based ILs (**c**) (0–1% *v*/*v*; 24h). The cell viability of ILs-exposed cells was evaluated by MTT assay. Values represent mean ± SD (n = 2–4), and are expressed as percentages of the non-treated control cells. IC_50_ values for each IL were calculated based on the viability data and expressed as a percentage (% *v*/*v*).

When considering the general cytotoxicity, the results showed that all the ILs (up to 1%) induced a concentration-dependent decrease of HaCaT cells viability (Figure 2a–c). Nonetheless, a different impact on cell viability was observed when HaCaT cells were exposed to different cation/anion IL combinations.

Globally, as observed in Figure 2, the viability of the HaCaT cells was less affected in the presence of the salts containing the cholinium cation. For the choline amino acid ILs, [Cho][Phe] and [Cho][Gly], a significant concentration-dependent decrease of HaCaT cell viability was observed (Figure 2a). These results also showed that, in general, imidazole-based ILs were more cytotoxic to HaCaT cells than the choline amino acid-based ILs (Figure 2b,c).

Some of these results are in accordance with a previous study from our group, in which some of these ILs were preliminary studied [23]. Herein, it was possible to perform a broader and integrated study and include [Cho][Br] salt, [Cho][Gly] IL, and the imidazole amino acid ILs ([Emim][Phe], [Bmim][Phe], [Emim][Gly], and [Bmim][Gly]).

Through the cytotoxic profile of the different ILs, it was possible to calculate IC_50_ values to better compare their impact on HaCaT cells. For the choline amino acid ILs, [Cho][Phe] and [Cho][Gly], a significant concentration-dependent decrease of HaCaT cell viability was observed, with the IC_50_ values of 0.47% and 0.65%, respectively (Figure 2a). This suggests that the glycinate anion may contribute to the lower toxicity of choline-derived IL. For the imidazole halogenated ILs, the IC_50_ values obtained were 0.57% for [Emim][Br] and 0.38% for [Bmim][Br] (Figure 2b). For the imidazole amino acid ILs, the IC_50_ values were 0.46% for [Emim][Phe], 0.34% for [Emim][Gly], 0.24% for [Bmim][Phe], and 0.34% [Bmim][Gly] (Figure 2c). In general, an increase in cytotoxicity was observed with the length of the alkyl chains (Figure 2b,c). This overall outcome may be explained, at least partly, by the change in surfactant properties provided by cations with longer alkyl chains, which can disrupt cell membrane integrity [36].

Thus, our results showed that the toxicity of ILs depends on both the cation and anion present in the IL, in fact the cation head group, the length of the cation side chain, and the anion all played important roles in the global toxicity of ILs.

Furthermore, and considering the main goal of this study, these results also allowed us to define the upper safer concentration of each IL to be used in further experiments.

The data revealed that to ensure the maintenance of cell viability, the highest concentration of ILs to be used in further studies should be 0.2% (*v*/*v*) for most of the prepared ILs. For [Cho][Gly] IL, it may be 0.5% (*v*/*v*), and for [Bmim][Br] IL and [Bmim][Phe] IL it should be 0.1% (*v*/*v*).

Hence, our results express that, in terms of ensuring safety, the greatest amount of IL to be included in topical formulations did not differ much amongst the prepared ILs.

Knowing this, the subsequent step was to evaluate the possible functionalities of each IL at these concentrations. Thus, since the increase in drug solubility is one of the most referenced applications of ILs in drug delivery, next, the impact of the eight prepared ILs on the drug solubility of four poorly water-soluble phenolic compounds was studied.

### 2.3. Influence of ILs on the Solubility of Poorly Soluble Phenolic Compounds (Ferulic, Caffeic, and p-Coumaric Acids, and Rutin)

Choline-based ILs have shown potential as solubility enhancers, improving the solubility of various poorly soluble compounds, such as ferulic acid and rutin [4,6].

Herein, to understand the impact of the eight prepared ILs on the solubility of ferulic, caffeic, and *p*-coumaric acids, as well as rutin, solubility studies were performed in water, in water:[Cho][Br] mixtures, and in water:IL mixtures, at 25 °C. Namely, at this point, we intended to understand if a combination between the imidazole-based cations and the amino acid-derived anions could represent an added value in terms of drug solubility.

First, several percentages of the salts were studied to understand the influence of their concentration on the solubility of each studied phenolic compound. The range of IL concentrations used (0.01%, 0.1%, 0.2%, 0.3%, and 0.5%) were selected according to the previously discussed cytotoxic results (Section 2.2). The solubility data of each phenolic compound, in water, and in the presence of water:[Cho][Br] and water:IL, are presented in Table 2.

The obtained solubilities for ferulic, caffeic, and *p*-coumaric acids in water were 0.63 mg/mL, 0.45 mg/mL, and 0.70 mg/mL, respectively, all below 1 mg/mL, which were in agreement with previously published results [6,37,38]. For rutin, the obtained solubility was even smaller, at 0.20 mg/mL; also in agreement with the literature [6] (Table 2).

Concerning the impact of the studied salts on drug solubility, our results showed that the imidazole halogenated ILs (Emim][Br], [Bmim][Br]) and the [Cho][Br] salt did not impact the drug solubility of the studied phenolic compounds.

On the other hand, at most concentrations of the remaining ILs, the amino acid ILs ([Cho][Phe], [Cho][Gly], [Emim][Phe], [Bmim][Phe], [Emim][Gly], and [Bmim][Gly]), were all effective in increasing the solubility of the four poorly water-soluble compounds under study. In fact, even at low concentrations of these ILs (0.1%), an enhancement in solubility was already attainable, in most cases. Additionally, when the percentage of these ILs increases, the solubility of the active compounds also rises, showing that a higher amount of IL allows a greater improvement in drug solubility.

In previous works, our group studied the impact of some choline-based ILs on the solubility of rutin and ferulic acid [4,6], but herein a broader range of concentrations was considered and this evaluation was extended to caffeic and *p*-coumaric acids. Additionally, it should also be noted that, to the best of our knowledge, it is the first time that solubility studies of these four phenolic compounds are presented in the presence of the six imidazole-based ILs ([Emim][Br], [Emim][Phe], [Emim][Gly], [Bmim][Br], [Bmim][Phe] and [Bmim][Gly]) and the salt [Cho][Br].

Looking at the overall solubility results, our data revealed that different combinations of cation/anion within the ILs affect the solubilizing ability, with the anions presenting a higher impact. For instance, when comparing the solubilities of the four phenolic compounds in the presence of [Emim][Br] with their solubilities in the presence of [Emim][Phe] and [Emim][Gly], it is quite clear that the first IL did not have an impact on the solubility, while at most concentrations the imidazole amino acid ILs both significantly increased the drug solubility. The same tendency was observed when comparing [Bmim][Br] with [Bmim][Phe] and [Bmim][Gly]. This clearly reinforces that the cation does not seem to be determinant for drug solubility; in opposition to the anion, which seemed to be the major contributor to the observed increase in the solubility. These results are in agreement with the literature, which previously suggested that anions seem to have more impact on the solubility than cations; this feature has been attributed to the ability that the anions have to form hydrogen bonds with the drug molecules [39].

Additionally, it was possible to perceive that the ILs containing the glycinate anion generally led to a higher solubility enhancement, compared to the ILs with the phenylalaninate anion. This was particularly prominent amongst the imidazole amino acid ILs, where at a concentration of 0.2% or above, the imidazole glycinate ILs always showed a higher drug solubility compared to the corresponding imidazole phenylalaninate ILs. Maybe the lower lipophilicity from the glycinate anion could justify this result, by facilitating drug solubility in the water:IL mixtures. Hence, even though it seems clear that anions have a more dominant impact on drug solubility, our results further showed that, when combined with the amino acid anions, cations have some influence on this parameter.

For example, for other drugs, namely salicylic acid and caffeine, we previously showed that choline-amino acid ILs were more suitable as solubility promotors than the imidazole-halogenated ILs [23]. Herein, we encountered the same behavior for the phenolic compounds studied, but our present results allowed a broader conclusion, since they showed that this difference was due to the anion present in the ILs, and not because the cation was choline-derived or imidazole-derived. In fact, the present study unveiled that, while in some cases the ILs with a choline cation allowed a slightly higher solubility enhancement, compared to the imidazole amino acid ILs, this was not always true. Namely, when comparing the results obtained in the presence of equal concentrations of [Cho][Gly] and [Bmim][Gly], in this case it was the latter that in general led to a greater drug solubility. Therefore, our study shows that imidazole-based ILs may also be used to enhance drug solubility if the incorporated anion is well chosen, and may even lead to a higher improvement in solubility. More precisely, combining the amino acid-derived anion that in general allowed the highest drug solubility (Gly anion) with the cation containing the longest side chain (Bmim anion) led to a greater impact on solubility and might be an interesting strategy to further exploit.

Finally, when considering pharmaceutical applications, namely by a topical route, it becomes crucial to cross reference the results related to functionality with the data that emerged from the cytotoxic studies, namely the upper concentration of each IL that may be used, while ensuring safety. In fact, when we analyzed the functionality of each IL as a solubility promoter, at non-toxic concentrations, and amongst the three different types of ILs studied, namely the imidazole halogenated ILs, the choline amino acid ILs, and the imidazole amino acid ILs, we concluded that the first type was clearly the least suited for topical applicability. This was justified by our results, which showed that the halogenated ILs had no impact on drug solubility.

On the other hand, all the amino acid ILs presented a clear impact on drug solubility at concentrations which allow the maintenance of cell viability.

Furthermore, and also in terms of functionality, it has been shown that the imidazole-based ILs have a higher impact on drug permeation (due to their alkyl side chain) when compared to the choline-based ILs [23]. Thus, this is another argument that highlights that combining the imidazole cations with the amino acid anions, while not neglecting the overall toxicity of the resulting ILs, could be quite a useful strategy when considering topical applications.

Thus, bearing all of this in mind, our next aim was to develop O/W emulsions and assess the impact of the prepared ILs on these formulations, particularly from a pharmaceutical technology point of view.

### 2.4. Oil-in-Water (O/W) Emulsions

The O/W emulsions were prepared by mixing the water and oily phases, each prepared according to the compositions described in Table 3. At first, the formulations were developed in the presence and absence of each IL, but without the phenolic compounds, to establish the impact of the ILs on the main formulation. Then, formulations containing each phenolic acid or rutin were also prepared in the absence and presence of ILs.

Since all the components included in a formulation can affect the stability of a newly developed system, and be a clear limiting factor, the impact of incorporating new materials, and the different concentrations of these components, always need to be evaluated. Thus, stability studies on all the developed emulsions were performed. The stability parameters evaluated included the organoleptic properties (appearance, color, and odor) and the physical–chemical properties, pH, and viscosity. This evaluation was performed after the formulation procedure (after 24 h) and then after submitting the emulsions to the stability studies. The preliminary stability studies performed involved centrifuging and gradual heating assays, followed by six temperature cycles [40].

After preparation of the formulations without the drug, all the emulsions were stable and odorless, and presented a white color and fluid appearance. The pH was adjusted to a pH equal to 5 for all the delivery systems, since the reported values for skin pH are within the acidic range for healthy skin [41].

Afterwards, stability studies were first performed on the emulsions prepared in the absence of the drugs. For comparison purposes, and since the upper concentration that could be safely used for most of the ILs was 0.2% (as assessed in Section 2.2), the emulsions were prepared containing this percentage of each IL (Table 4).

Following the preliminary stability studies, our results showed that the control formulation without any IL (Table 4, control 1) became unstable, presenting phase separation. Moreover, what was more remarkable was the fact that the formulations in the presence of all the studied ILs proved to be stable after the performed stability studies. This indicates that the studied ILs may act as stabilizers and increase the stability of the emulsions, which is quite a valuable functionality when developing emulsions, since even though these types of formulation are quite advantageous, they are thermodynamically unstable systems [42], and finding ways to overcome this challenge is crucial.

Moreover, another property evaluated herein that may be quite relevant when determining the performance of an O/W emulsion is the viscosity. This analysis is helpful to detect non-visible alterations that could impact the stability and/or the sensorial perceptions of the formulations. Hence, the viscosity of the emulsions was assessed, after their preparation and then again after the stability studies, for all the formulations that maintained stability.

Our results clearly showed that the ILs had an impact on this parameter (Table 4). After the performed stability studies, the data showed that different ILs presented different effects on the viscosity of the formulations and, interestingly, the amino acid ILs conveyed a higher viscosity to the emulsions compared to the halogenated salts. Maybe the amino acid anions have a higher tendency to participate in hydrogen bonding, which could lead to a higher viscosity [43]. Furthermore, the increase in viscosity could reduce the mobility of the droplets in the emulsion, preventing them from coalescing, which may avoid phase separation [44].

Thus, besides allowing the higher stability of the prepared O/W emulsions, the studied ILs may also be valuable for adjusting the viscosity of the formulations. This could be another useful function conferred by the ILs, since altering the viscosity is quite relevant, not only in terms of stability, but also because the sensory properties, which depend on viscosity, are essential for ensuring the consumer’s acceptance of the developed product [45].

Hence, ILs could be useful to attain more efficient O/W emulsions by allowing a fundamental balance between the stability of the system and the sensory properties, both characteristics fundamental to developing a high-quality formulation.

Following this, since the ILs derived from glycinate generally presented a lower toxicity and allowed a higher enhancement in drug solubility compared with phenylalaninate derived ILs, the three glycinate ILs ([Cho][Gly], ([Emim][Gly], and ([Bmim][Gly]) were further studied as functional excipients for the inclusion of the four phenolic compounds under study.

For comparison purposes, the O/W emulsions were also prepared using an equal amount of each glycinate IL (0.2%) (Table 5). Since for [Cho][Gly] the upper concentration that could be used safely is 0.5%, in this case, two emulsions containing this IL, at 0.2% and 0.5% were prepared. For all the developed formulations, the phenolic compounds were always included at the maximum concentration that each of them was soluble in the percentage of IL that was included in the formulation. This was performed considering the solubility results previously obtained in this study (Table 2). Additionally, emulsions containing each phenolic compound, but without the ILs (Table 5, Controls 2a–d), were also prepared, but now including the upper concentration of each phenolic compound that is soluble in water alone, since in this case, no IL is present to allow a higher drug solubility.

When comparing the formulations containing the phenolic compounds, it was possible to conclude that the O/W emulsions containing ILs were easier to prepare, indicating that the ILs not only allowed a higher drug loading, but also facilitated the incorporation of the studied actives.

Moreover, after formulation all the emulsions were stable, with a homogeneous appearance. Their color was uniform for each drug included, with the emulsions containing rutin presenting a slightly yellow appearance, while the ones containing the phenolic acids presented an off-white color. All formulations were odorless, and their pH was adjusted to 5.

The control formulations (in the absence of IL, Controls 2a–d) again proved to be unstable, after the accelerated stability studies, while the formulations containing IL and each phenolic compound were all stable after the stability studies (Table 5). This corroborated the hypothesis that the studied ILs acted as stabilizers of the developed emulsions, with this being true both in the absence and in the presence of the phenolic compounds under study (Table 4 and Table 5).

It should also be mentioned, that besides stabilizing the topical systems, these ILs allow the incorporation of much higher amounts of each phenolic compound, due to their increased solubility in the presence of these materials. Higher drug loading was achieved in the presence of the [Bmim][Gly] IL, at 0.2%, and the [Cho][Gly] IL, at 0.5%.

Finally, the impact of the studied ILs on the stability of the formulations over time was also studied, for 90 days [40]. In this analysis, the emulsions were submitted to less extreme conditions compared to the preliminary stability tests, allowing the prediction of the expiration date. All the samples were submitted to heating in an oven (40 ± 2 °C) and cooling in a refrigerator (5 ± 2 °C).

Samples of all the prepared emulsions that maintained stability after the preliminary stability assays were also stored at room temperature to evaluate their shelf life and long-term stability, and these formulations were macroscopically evaluated over time. After 90 days, all the studied batches, from the three different conditions, were evaluated in terms of their organoleptic properties, pH, and viscosity (Table 6 and Table 7).

For all the studied formulations, either without (Table 6) or containing each drug (Table 7), no signs of instability were observed. This suggests that the ILs may be efficient at ensuring the stability of the formulations, not only upon formulation or after being submitted to the extreme conditions of the preliminary stability studies, but also over time (for a period equivalent to 3 months). Additionally, all the formulations presented a pH between 4.8 and 5.0, which is suitable for a topical emulsion, as previously mentioned. In terms of viscosity, all the formulations presented a similar tendency, as observed after the preliminary stability studies, showing that the salts containing a smaller anion lead to smaller viscosities. Moreover, our results also indicated that the choline ILs seem to lead to a greater increase in viscosity over time, compared with imidazole ILs. Nonetheless, all the emulsions continued to be stable and present a fluidity suitable for this type of formulation.

Overall, our study reinforced the relevance of including ILs in topical delivery systems and reveals that their functionality may in fact be multiple. It particularly stressed that choosing the right combination of cation/anion, while carefully considering the upper concentration of each IL that can safely be used, might be a key strategy to take full advantage of these materials in drug delivery. Namely, ILs may not only enhance the solubility of various poorly soluble drugs and improve their incorporation into O/W emulsions, but may also facilitate the formulation procedure and act as stabilizers of the developed emulsions, while allowing the use of lower concentrations of emulsifier, and may even be useful to alter the viscosity of the formulations.

## 3. Materials and Methods

### 3.1. Equipment and Chemicals

The studied actives were ferulic acid from Henrifarma (São Paulo, Brazil), *p*-coumaric and caffeic acids acquired from Sigma-Aldrich (Saint Louis, MO, USA), and rutin, which was purchased from Fragon (São Paulo, Brazil).

For the ILs synthesis, the reagents and solvents used from the Sigma-Aldrich supplier (Saint Louis, MO, USA) were choline hydroxide in methanol [Cho][OH]/MeOH 45%, methanol, Amberlite^®^ IRA-400 chloride form, 1-bromoethane, potassium hydroxide (KOH), choline bromide, and glycine. Then from the Fluka supplier (Seelze, Germany) was used 1-bromobutane, from Alfa Aesar Chemicals (Haverhill, MA, USA) the 1-methylimidazole, from VWR (Fontenay-sous-Bois, France) the acetonitrile, and from PanReact AppliChem (Barcelona, Spain) the sodium hydroxide (NaOH) and the lphenylalanine. The reagents used for the cell viability studies were fetal bovine serum (FBS) and Dulbecco’s modified Eagle’s medium (DMEM) acquired from Biowest (Nuaillé, France). The phosphate buffered saline (PBS; 0.01 M, pH 7.4), trypsin, penicillin–streptomycin solution, dimethyl sulfoxide (DMSO), and thiazolyl blue tetrazolium bromide (MTT) were obtained from Sigma-Aldrich (Saint Louis, MO, USA). All the IL solutions used for the different tests were prepared using sterile water.

The synthesized ILs were identified by ^1^H NMR, ^13^C NMR, and FTIR spectra. ^1^H NMR and ^13^C NMR were acquired using a Brucker Avance 400^®^ apparatus (Billerica, MA, USA) at 400 and 100.4 MHz, respectively, using D_2_O or DMSO as solvents. The chemical shifts are expressed in parts per million (ppm, δ). The following abbreviations in ^1^H-NMR were used to designate multiplicities: s = singlet, d = doublet, t = triplet, q = quartet, m = multiplet, dd = double-doublet.

The IR spectra were recorded on a PerkinElmer^®^ Spectrum (Waltham, MA, USA) equipped with an attenuated total reflectance (ATR) device. The spectra were obtained collecting 100 scans of each sample with a resolution of 4 cm^−1^, between 4000 and 450 cm^−1^.

In the development of the oil-in-water (O/W) emulsions, the compounds were divided into two phases. The oily phase was composed of Crodafos^®^ CES (cetearyl alcohol (and) dicetyl phosphate (and) ceteth-10 phosphate) (Mapric^®^, São Paulo, Brazil), the isopropyl myristate (Scharlab, Sentmenat, Spain), and the butylated hydroxytoluene, BHT, (Mapric^®^, São Paulo, Brazil). On the other hand, the aqueous phase constituted ethylenediaminetetraacetic acid disodium dihydrate, EDTA Na_2_, (Fragon, Barcelona, Spain), propylene glycol (Fragon, Barcelona, Spain), polyethylene glycol 400 (PanReact, Barcelona, Spain), methylparaben and propylparaben (Sigma-Aldrich, Saint Louis, MO, USA), and triethanolamine (José Vaz Pereira, Lisbon, Portugal).

### 3.2. Synthesis of Ionic Liquids

The prepared ILs were synthetized through various methodologies, described below for each IL. All ILs were characterized by ^1^H NMR, ^13^C NMR, and FTIR (Supplementary Materials).

#### 3.2.1. 2-hydroxyethyl-trimethylammonium-l-phenylalaninate [Cho][Phe] (**1**), 2-hydroxyethyl-trimethylammonium glycinate [Cho][Gly] (**2**).

The choline amino acid-based ILs, (**1**) and (**2**), were synthesized according to previously described procedures [12,23]. Briefly, a solution of choline hydroxide in methanol [Cho][OH]/MeOH 45% (57.76 mmol) was evaporated under vacuum at 50–60 °C to remove methanol. Then, an excess of the corresponding amino acid (57.79 mmol), either lphenylalanine or glycine, was solubilized, in the minimum amount of water, and added to the previous solution. The blend was then cooled in an ice bath and stirred overnight. Next, the water was removed under vacuum at 60 °C, and a mixture of acetonitrile and methanol (9:1) was added to precipitate the unreacted amino acid. The mixture was stirred vigorously and then centrifuged (at 1500 rpm for 30 min) and filtered. Finally, the solvents were evaporated under vacuum at 60 °C, and the obtained IL was stored until use under moisture-free conditions.

(**1**) Orange viscous liquid, ƞ = 83%; **^1^H NMR** (D_2_O) δ (ppm) 2.73 (dd, 1H, J_1_ = 7.26 Hz, J_2_ = 13.45 Hz, H-3a’), 2.87 (1H, J_1_ = 5.77 Hz, J_2_ = 13.45 Hz, H-3′b), 3.06 (s, 9H, N-(CH_3_)_3_), 3.35–3.39 (m, 2H, H-2 and 1-H, H-2′), 3.90–3.93 (m, 2H, H-3), 7.15–7.28 (m, 5H, H-Ph). **^13^C NMR** (D_2_O) δ (ppm) 40.68 (C-2′, CH_2_), 53.74, 53.78, 53.82 (N-(CH_3_)_3_, CH_3_), 55.54 (C-3, CH_2_), 57.40 (C-3′, CH_2_), 67.42 (C-2, CH_2_), 126.60, 128.54, 129.39 (C-Ph’, CH), 138.22 (C-Ph’, Cq), 182.32 (C-1′, Cq). **FTIR** $\bar{\upsilon }$ (cm^−1^): 861.0, 1084.0, 1399.0, 1475.2, 1556.3, 2928.8, 3428.4.

(**2**) Yellow viscous liquid, ƞ = 92%; **^1^H NMR** (D_2_O) δ (ppm) 3.02 (s, 2H, H-2′), 3.04 (s, 9H, N-(CH_3_)_3_), 3.35 (t, 2H, *J* = 6.0 Hz, H-2), 3.87–3.90 (m, 2H, H-3). **^13^C NMR** (D_2_O) δ (ppm) 44.40 (C-2′, CH_2_), 53,74, 53.78, 53,82, (N-(CH_3_)_3_, CH_3_), 55.49 (C-3, CH_2_), 67.34 (C-2, CH_2_), 180.60 (Cq-1′). **FTIR** $\bar{\upsilon }$ (cm^−1^): 889.0, 1085.0, 1396.0, 1476.0, 1568.4, 2957.0, 3441.7.

#### 3.2.2. 1-ethyl-3-methylimidazolium bromide [Emim][Br] (**3**), 1-butyl-3-methylimidazolium bromide [Bmim][Br] (**6**)

The imidazolium-based ILs (**3**), and (**6**), were prepared according to previously described procedures with slight modifications [12]. Briefly, under vigorous stirring, an excess of bromoethane or 1-bromobutane (135 mmol) was added dropwise over 15 min to 1-methylimidazol (45 mmol) and then were stirred overnight at room temperature. For each synthesis the corresponding unreacted bromoalkane (bromoethane or 1-bromobutane) was evaporated under vacuum. Then, the respective ILs obtained, (**3**) or (**6**), were stored under moisture-free conditions until use.

(**3**) White solid, ƞ = 99%; m.p. = 63–65 °C;**^1^H NMR** (D_2_O) δ (ppm) 1.39 (t, 3H, *J* = 7.2 Hz, H-7), 3.79 (s, 3H, N-CH_3_), 4.12 (q, 2H, *J* = 7.2 Hz, H-6), 7.33 (s, 1H, H-4 or H-5), 7.39 (s, 1H, H-4 or H-5), 8.63 (s, 1H, H-2). **^13^C NMR** (D_2_O) δ (ppm) 14.61 (C-8, CH_3_), 35.77 (N-CH_3,_ CH3), 44.83 (C-7, CH_2_), 121.90, 123,47 (C-4 and C-5, CH), 135.59 (C-2, CH). **FTIR** $\bar{\upsilon }$ (cm^−1^): 621.4, 750.8, 842.4, 1168.4, 1448.1, 1569.5, 2975.0, 3063.1.

(**6**) White solid, ƞ = 72%; m.p. = 64–66 °C;**^1^H NMR** (D_2_O) δ (ppm) 0.81 (t, 3H, *J* = 7.2 Hz, H-9), 1.23–1.18 (m, 2H, H-8), 1.76–1.72 (m, 2H, H-7), 3.79 (s, 3H, N-CH_3_), 4.09 (t, 2H, *J* = 7.2 Hz, H-6), 7.33 (s, 1H, H-4 or H-5), 7.38 (s, 1H, H-4 or H-5), 8.63 (s,1H, H-2). **^13^C NMR** (D_2_O) δ (ppm) 12.63 (C-9, CH_3_), 18.73 (C-8, CH_2_), 31.23 (C-7, CH_2_), 35.63 (N-CH_3_, CH3), 49.24 (C-6, CH_2_), 122.14, 123.39 (C-4 and C-5, CH), (C-4, CH), 131.70 (C-2, CH). **FTIR** $\bar{\upsilon }$(cm^−1^): 619.3, 750.6, 837.0, 1167.3, 1460.1, 1575.3, 2868.0, 2931.2, 2957.1, 3067.1.

#### 3.2.3. 1-ethyl-3-methylimidazolium lphenylalaninate [Emim][Phe] (**4**)

The 1-ethyl-3-methylimidazolium lphenylalaninate, [Emim][Phe] (**4**) was prepared according to a previously described protocol [12] with slight modifications. Briefly, the intermediate 1-ethyl-3-methylimidazolium hydroxide [Emim][OH] was prepared reacting [Emim][Br] (21 mmol) with KOH (22 mmol) in methanol. The reaction mixture was stirred for 12 h at 60 °C. The formed KBr was removed. Then120 mL of water was added to the solution of [Emim][OH] thus prepared, and methanol was evaporated under vacuum at 60 °C. Next, the amino acid (21 mmol), solubilized in a minimum amount of water, was added dropwise to the previous solution and the resulting mixture was then stirred vigorously overnight at room temperature. Then, the water was removed under vacuum at 60 °C and the obtained IL was stored until use under moisture-free conditions.

(**4**) Yellow viscous liquid, ƞ = 67%; **^1^H NMR** (DMSO) δ (ppm) 1.39 (t, 3H, *J* = 7.2 Hz, H-7), 2.44 (m, 1H, H-3a’), 3.01 (dd, 1H, *J* = 13.55 Hz, H-3b’), 3.06–3.14 (m, 1H, H-2′), 4.18 (q, 2H, *J* = 7.2 Hz, H-6), 7.20–7.13 (m, 5H, H-Ph), 7.77 (s, 1H, H-4 or H-5), 7.69 (s, 1H, H-4 or H-5), 9.34 (s, 1H, H-2). **^13^C NMR** (DMSO) δ (ppm) 15.60 (C-7, CH_3_), 36.08 (NCH_3_, CH_3_), 42.47 (C-3′, CH_2_), 44.53 (C-6, CH_2_), 58.30 (C-2′, CH), 122.34, 123.95 (C-4 and C-5, CH), 129.77, 128.28, 125.88 (C-Ph’, CH), 136.96 (C-2, CH), 141.32 (C-Ph’, Cq), 177.37 (C-1′, Cq). **FTIR** $\bar{\upsilon }$(cm^−1^): 617.2, 743.0, 824.0, 1010.2, 1167.0, 1332.4, 1399.9, 1452.3, 1494.2, 1563.3, 2938.7, 3145.1, 3348.1.

#### 3.2.4. 1-ethyl-3-methylimidazolium glycinate, [Emim][Gly] (**5**), 1-butyl-3-methylimidazolium l-phenylalaninate [Bmim][Phe] (**7**) and 1-butyl-3-methylimidazolium glycinate [Bmim][Gly] (**8**)

The method used to prepare the ILs **5**, **7**, and **8** consisted in using anion exchange resin. To prepare the precursors, [Emim][OH] or [Bmim][OH], a chromatographic column containing an anion exchange resin (Amberlite^®^ IRA-400 chloride form) was prepared in NaOH (1 M) and then washed with NaOH (0.5 M) and then water until reaching neutrality. Next, the imidazolium halogenated based IL (10.47 mmol of [Emim][Br] to obtain compound **5** or 9.12 mmol of [Bmim][Br] for compounds **7** and **8**) was dissolved in deionized water, added to the column, and then several fractions were collected until the pH of the collected fractions was neutral. Next, for each IL, the combined collected fractions were added dropwise to an aqueous solution containing a molar excess of the respective amino acid (glycine to obtain compounds **5** and **8,** or phenylalanine to prepare the IL **7**), which had been previously solubilized in the minimum amount of water. The resulting mixture was then cooled in an ice bath and stirred overnight. The water was removed under vacuum at 60 °C and acetonitrile was added to precipitate the unreacted amino acid. The mixture was stirred vigorously and then centrifuged (at 450 × *g* for 20 min) and filtered. Finally, the solvent was evaporated under vacuum at 60 °C and the ILs obtained in each case (**5**, **7,** and **8**) were stored until use under moisture-free conditions.

(**5**) Light yellow liquid, ƞ = 65%; **^1^H NMR** (DMSO) δ (ppm) 1.40 (t, 3H, *J* = 7.38Hz, H-7), 2.74 (s, 2H, H-2′), 3.87 (s, 3H, N-CH_3_), 4.22 (q, 2H, *J* = 7.2 Hz, H-6), 7.76 (s, 1H, H-4 or H-5), 7.85 (s, 1H, H-4 or H-5), 9.61 (s, 1H, H-2). **^13^C NMR** (DMSO) δ (ppm) 15.66 (C-7, CH_3_), 36.03 (NCH_3_, CH_3_), 44.46 (C-6, CH_2_), 46.60 (C-2′, CH_2_), 122.39, 123.97 (C-4 and C-5, CH), 137.32 (C-2, CH), 175.81 (Cq, C-1′). **FTIR** $\bar{\upsilon }$ (cm^−1^): 698.0, 742.0, 883.6, 1005.6, 1111.2, 1169.8, 1390.2, 1403.2, 1451.1,1566.0, 2982.1, 3154.0, 3361.1.

(**7**) Yellow viscous liquid, ƞ = 62%; **^1^H NMR** (DMSO) δ (ppm) 0.89 (t, 3H, *J* = 8 Hz, H-9), 1.22–1.27 (m 2H, H-8), 1.74–1.77 (m, 2H, H-7), 2.46 (t, 1H, H-2′, *J* = 10.81Hz,), 3.02 (d, 2H, *J* = 13.2Hz, H-3′), 3.86 (s, 3H, N-CH3), 4.17 (t, 2H, *J* = 8Hz, H-6), 7.20–7.13 (m, 5H, H-Ph’), 7.74 (s, 1H, H-4) 7.81 (s, 1H, H-5), 9.51 (s, 1H, H-2). **^13^C NMR** (DMSO) δ (ppm) 13.75 (C-9, CH3), 19.24 (C-8, CH2), 31.86 (C-7, CH2), 36.11 (N-CH3, CH3), 42.55 (C-3′, CH2), 48.86 (C-6, CH2), 58.26 (C-2′, CH), 122.67 (C-5, CH), 124.02 (C-4, CH), 125.83, 128.28, 129.72 (C-Ph’, CH), 137.47 (C-2, CH), 141.57 (C- Ph’, Cq), 176.53 (C-1′). **FTIR** $\bar{\upsilon }$ (cm-1): 620.1, 744.0, 820.4, 1170.2, 1386.7, 1453.4, 1494.2, 1562.3, 2871.6, 2931.1, 2958.0, 3092.2, 3143.0.

(**8**) Light yellow viscous liquid, ƞ = 65%; **^1^H NMR** (DMSO) δ (ppm) 0.87 (t, 3H, *J* = 8 Hz, H-9), 1.20–1.25 (m, 2H, H-8), 1.71–1.78 (m 2H, H-7), 2.74 (s, 2H, H-2′), 3.88 (s, 3H, N-CH_3_), 4.20 (t, 2H, *J* = 8Hz, H-6), 7.82 (s, 1H, H-4) 7.88 (s, 1H, H-5), 9.84 (s, 1H, H-2). **^13^C NMR** (DMSO) δ (ppm) 13.44 (C-9, CH_3_), 19.02 (C-8, CH_2_), 31.52 (C-7, CH_2_), 35.98 (N-CH_3_, CH_3_), 45.34 (C-2′, CH_2_), 49.13 (C-6, CH_2_), 122.44 (C-5, CH), 123.77 (C-4, CH), 136.19 (C-2, CH), 178.36 (C-1′-Cq). **FTIR** $\bar{\upsilon }$ (cm^−1^): 623.8, 733.0, 881.0, 1169.0, 1390.8, 1462.2, 1562.0, 2877.2, 2941.1, 2960.5, 3095.4, 3153.7.

### 3.3. Cell Culture

HaCaT (human keratinocytes) cells were cultured in DMEM medium supplemented with 10% FBS, 100 U/mL penicillin, and 0.1 mg/mL streptomycin. Cells were maintained at 37 °C, under a humidified air atmosphere containing 5% of CO_2_.

### 3.4. MTT Assay/Cell Viability Assay

To evaluate the cytotoxicity of the synthetized ILs, cell viability was determined using the MTT reduction assay, adapted from previously published procedures [46,47]. Briefly, the HaCaT cells were seeded into 96-well plates at a density of 6 × 10^3^ per well in 200 µL culture medium and incubated for 24 h. Then, cells were exposed to different concentrations (0–1%) of each prepared IL for a 24 h period. After the treatment, the cells were rinsed with warm PBS and then MTT (dissolved in warm culture medium, at a 0.5 mg/mL concentration) was added to each well. Cells were then incubated for a further period of 3.5 h. Then, after rinsing the cells with warm PBS, 100% DMSO was added to each well. A Multiskan FC microplate reader (Thermo Scientific™, USA) was used to read the absorbance at a wavelength of 595 nm. The results were expressed as percentages relative to the control (which corresponded to 100% cell viability). For this assay, two to four independent experiments were carried out and in each independent experiment at least four replicate cultures were used.

The half-maximal inhibitory concentration (IC_50_) was assessed by GraphPad Prism 7^®^ Statistical Software (San Diego, CA, USA).

### 3.5. Solubility Studies

The solubility studies were carried out according to previously described protocols [4,6]. Briefly, different solutions containing water or water:IL mixtures (99.99:0.01 to 99.5:0.5%, *w*/*w*) were prepared in triplicate and saturated with each of the following four compounds: rutin, or ferulic, caffeic, or *p*-coumaric acids. Then, using a horizontal shaker (IKA VIBRAX VXR^®^, LTF Labortechnik GmbH & Co., Bodensee, Germany) the solutions were shaken for 72 h at 25 °C. Following this the solutions were filtered, to remove the solute that was in excess, and then studied using UV–visible spectrophotometry, on an Evolution^®^ 300 (Thermo Scientific, Hertfordshire, England). The analysis was performed at 286 nm for *p*-coumaric acid, 313 nm for ferulic and caffeic acids, and 353 nm for rutin (the corresponding maximum absorption wavelength of each studied compound in water).

### 3.6. Production of the Oil-in-Water (O/W) Emulsions

The O/W emulsions were prepared in the presence or absence of the synthetized IL, according to the qualitative and quantitative (%, *w*/*w*) compositions described in Table 3. All compounds were weighted separately for both phases, the oil and aqueous phase. The phases were heated at 65 °C in a water bath. When both phases reached the same temperature, the aqueous phase was added to the oil phase under stirring. Then, the formulations were stirred until they cooled to room temperature.

The organoleptic characteristics, pH, and viscosity of all formulations were analyzed 24 h after preparation and after performing the stability studies. The pH was measured with a pH meter 827 from Metrohm^®^, Herisau, Switzerland, and the viscosity was measured with a rheometer DV3T from AMETEK (Brookfield, Preston, United Kingdom).

### 3.7. Stability Studies of the O/W Emulsions

First, the centrifuge and the gradual heating assays were performed [6,48]. In the centrifugation test, 5 g of each formulation was heated at 45 °C for 30 min and then centrifuged for 30 min at 7200× *g*. Regarding the gradual heating or thermal stress, 5 g of each emulsion were weighed, which were then placed in a thermostatic bath, starting from an initial temperature of 40 °C increasing to a final temperature of 80 °C, with an increase of 10 °C every 30 min.

Then, the formulations were subjected to six temperature cycles [40,48]. Each cycle was completed by placing the product in the freezer (-5 °C) for 24 h and 45 °C for another 24 h. At the end of the six temperature cycles the organoleptic properties, the pH, and the viscosity were again assessed.

Accelerated and shelf-life stability tests were also performed [40]. First, the O/W emulsions were divided and submitted to two different conditions, oven (40 ± 2 °C) and refrigerator (5 ± 2 °C) for 90 days (3 months). For the shelf-life stability test, the third batch of all the emulsions was stored at room temperature and these formulations were macroscopically evaluated over time.

After 90 days, the organoleptic properties, pH, and viscosity of all the formulations, subdivided within the three referred conditions, were analyzed again.

For all stability studies, the samples were prepared in triplicate.

### 3.8. Statistical Analysis

The solubility results, which are expressed as mean ± standard deviation, were assessed with one-way analysis of variance (ANOVA) followed by Tukey’s multiple comparison test. The analysis was performed with SPSS^®^ (Statistical Package for the Social Sciences), version 22 (SPSS Inc. Chicago, IL, USA).

## 4. Conclusions

ILs have proven to be an interesting tool to be used in several areas, such as the pharmaceutical and cosmetic sectors. Moreover, since their valuable properties may confer them with various attractive uses, ensuring to take full advantage of their characteristics upon synthesis is quite relevant.

Hence, in this study, eight ILs were synthesized with different combinations of cation and anion within the ILs, to assess if these materials could lead to multiple functionalities in topical emulsions. At first, the upper concentration of each IL that could be used to ensure a safe applicability was determined in human keratinocytes HaCaT cells. Then, since poor drug solubility is a huge limitation upon the development of new drug delivery systems and impairs the use of several drugs with promising properties, such as ferulic, caffeic, and *p*-coumaric acids, as well as rutin, the impact of various concentrations of each IL on the solubility of these four phenolic compounds was evaluated. The results showed that the different combinations of cation/anion influenced the drug solubility, with the anions having more impact on this functionality. Moreover, the amino acid ILs proved to be the better solubility promotors, independently of the cation present being choline or imidazole derived. Amongst the studied amino acids, generally, the glycinate based ILs allowed a higher solubility enhancement.

Then the incorporation of the ILs in O/W emulsions, at nontoxic concentrations, was studied, and interestingly the results showed that they improved the performance of the formulations in multiple ways. The ILs not only allowed a considerably higher drug load of the four studied compounds, as a consequence of their impact on drug solubility, but they also proved to be a versatile technological strategy. In fact, they also improved the formulation procedure, the stability of the developed emulsions, and increased the viscosity of the formulations. Moreover, even though all the studied ILs showed an impact on these last parameters, this study also showed that the combination of the imidazole cation, which could be relevant for increasing drug permeation, with an amino acid anion (namely the glycinate, which generally ensures a greater increase in drug solubility), could be a successful strategy in topical drug delivery.

Therefore, the incorporation, and the right choice, of ILs may lead to an upgrade to multiple features of O/W emulsions, namely by improving the topical delivery of various poorly soluble drugs, the preparation procedure, and the stability of the formulations, while also being useful to tune the sensorial performance of the delivery systems.

## Figures and Tables

**Table 1:** Chemical structures, color, physical state at room temperature (RT), melting point (when applicable), and yields of synthetized ionic liquids (**1** to **8**).

IL			Color	Physical State at RT	Melting Point (°C)	Reaction Yield (%)
Acronym	Cation	Anion				
[Cho][Phe] (**1**)	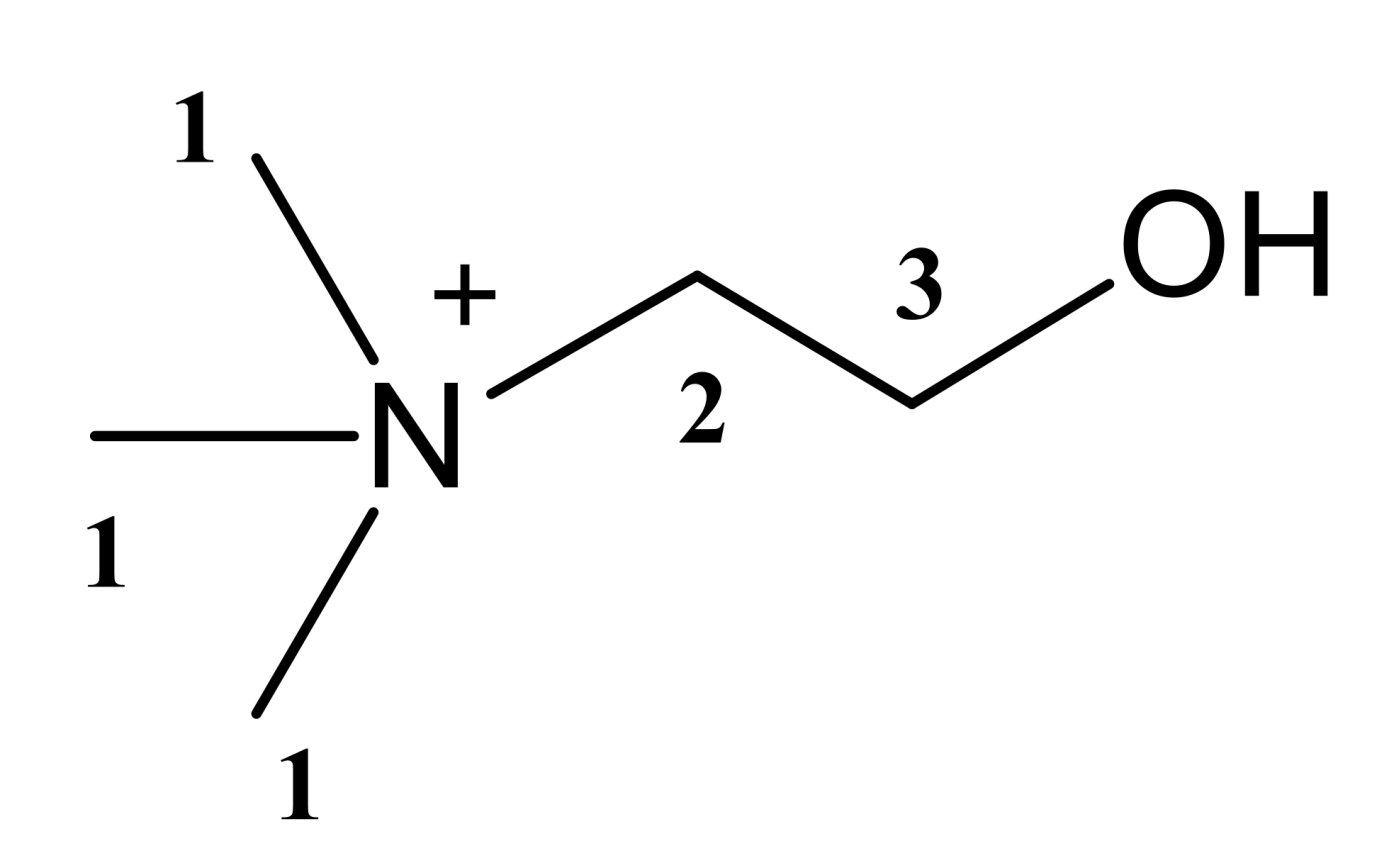	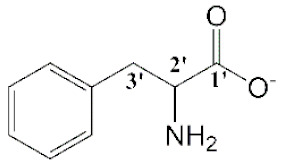	Orange	Viscous liquid	-	83
[Cho][Gly] (**2**)		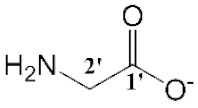	Yellow	Viscous Liquid	-	92
[Emim][Br] (**3**)	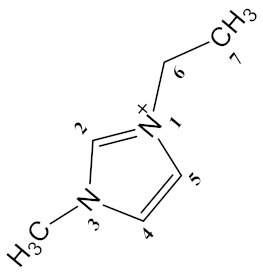	Br^−^	White	Viscous Solid	63-65	99
[Emim][Phe] (**4**)		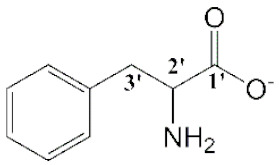	Yellow	Viscous Liquid	-	67
[Emim][Gly] (**5**)		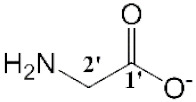	Light yellow	Viscous Liquid	-	65
[Bmim][Br] (**6**)	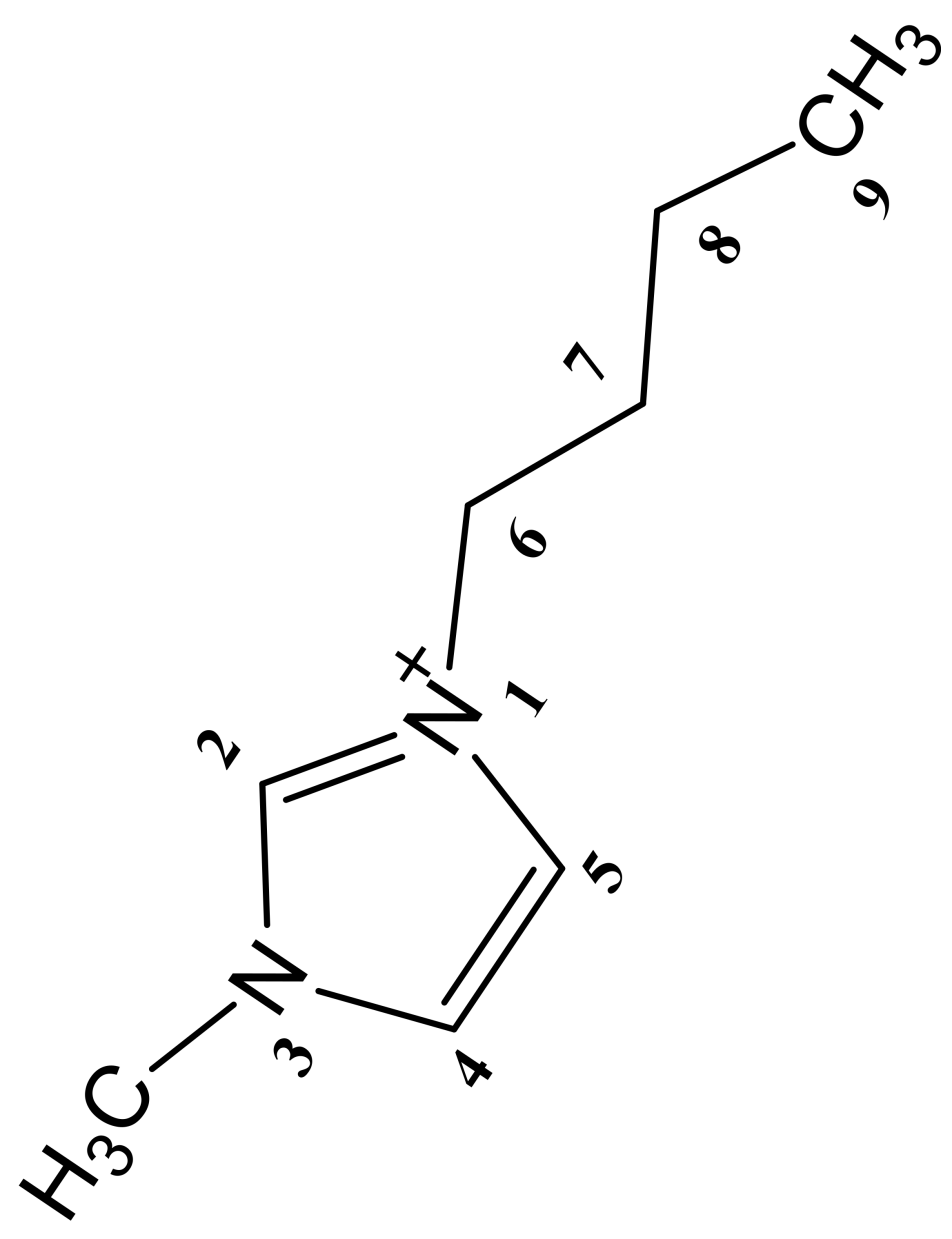	Br^−^	White	Viscous Solid	64–66	72
[Bmim][Phe] (**7**)		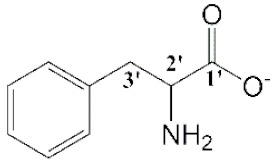	Yellow	Viscous Liquid	-	62
[Bmim][Gly] (**8**)		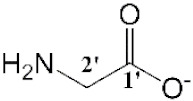	Light yellow	Viscous Liquid	-	65

**Table 2:** Solubility results for ferulic, caffeic, and *p*-coumaric acids, and rutin in deionized water and in different deionized water:IL mixtures at 25 ± 2 °C.

Solubility (mg/mL)				Solvent (% *w*/*w*)	
Ferulic Acid	Caffeic Acid	*p*-Coumaric Acid	Rutin		
0.63 ± 0.04 ^a^	0.45 ± 0.04 ^a^	0.70 ± 0.04 ^a–d^	0.20 ± 0.01 ^a^	100	Water
0.63 ± 0.02 ^a,b^	0.45 ± 0.04 ^a^	0.65 ± 0.01 ^a–c^	0.19 ± 0.01 ^a^	99.99:0.01	Water:[Cho][Br]
0.67 ± 0.01 ^a,b^	0.45 ± 0.02 ^a^	0.65 ± 0.01 ^a–c^	0.20 ± 0.01 ^a^	99.9:0.1	
0.65 ± 0.01 ^a,b^	0.45 ± 0.02 ^a^	0.61 ± 0.01 ^a,b^	0.18 ± 0.01 ^a^	99.8:0.2	
0.63 ± 0.02 ^a,b^	0.49 ± 0.02 ^a,b^	0.52 ± 0.05 ^a^	0.18 ± 0.01 ^a^	99.7:0.3	
0.63 ± 0.01 ^a^	0.55 ± 0.01 ^a–d^	0.52 ± 0.05 ^a^	0.18 ± 0.01 ^a^	99.5:0.5	
0.63 ± 0.04 ^a^	0.54 ± 0.06 ^a–d^	0.73 ± 0.01 ^b–d^	0.26 ± 0.01 ^a^	99.99:0.01	Water:[Cho][Phe]
0.85 ± 0.07 ^a–f^	1.02 ± 0.03 ^h^	0.88 ± 0.04 ^d–g^	0.78 ± 0.08 ^b^	99.9:0.1	
1.06 ± 0.01 ^e–h^	1.42 ± 0.08 ^i^	1.39 ± 0.02 ^i–k^	1.44 ± 0.10 ^e^	99.8:0.2	
1.52 ± 0.13 ^i,j^	2.00 ± 0.12 ^k,l^	1.58 ± 0.02 ^l,m^	2.49 ± 0.12 ^i^	99.7:0.3	
2.12 ± 0.19 ^m–o^	2.35 ± 0.06 ^m^	1.74 ± 0.02 ^m,n^	2.78 ± 0.15 ^j^	99.5:0.5	
0.65 ± 0.08 ^a,b^	0.52 ± 0.03 ^a–c^	0.73 ± 0.05 ^b–d^	0.24 ± 0.01 ^a^	99.99:0.01	Water:[Cho][Gly]
0.94 ± 0.06 ^c–f^	0.88 ± 0.04 ^e–h^	0.96 ± 0.12 ^f,g^	0.82 ± 0.05 ^b,c^	99.9:0.1	
1.23 ± 0.29 ^g,h^	1.39 ± 0.07 ^i^	1.54 ± 0.07 ^k,l^	1.73 ± 0.03 ^f–h^	99.8:0.2	
1.66 ± 0.21 ^j,k^	1.83 ± 0.03 ^j,k^	1.86 ± 0.13 ^n,o^	2.63 ± 0.06 ^i,j^	99.7:0.3	
2.25 ± 0.10 ^n,o^	2.35 ± 0.03 ^m^	2.09 ± 0.11 ^p^	2.83 ± 0.09 ^j^	99.5:0.5	
0.61 ± 0.01 ^a^	0.46 ± 0.02 ^a^	0.69 ± 0.02 ^a–c^	0.18 ± 0.02 ^a^	99.99:0.01	Water:[Emim][Br]
0.62 ± 0.01 ^a^	0.47 ± 0.01 ^a,b^	0.73 ± 0.04 ^b–d^	0.21 ± 0.01 ^a^	99.9:0.1	
0.64 ± 0.03 ^a,b^	0.49 ± 0.03 ^a,b^	0.75 ± 0.06 ^b–e^	0.21 ± 0.02 ^a^	99.8:0.2	
0.64 ± 0.06 ^a,b^	0.51 ± 0.05 ^a,b^	0.74 ± 0.04 ^b–d^	0.24 ± 0.01 ^a^	99.7:0.3	
0.70 ± 0.01 ^a–c^	0.54 ± 0.01 ^a–d^	0.80 ± 0.02 ^c–f^	0.26 ± 0.02 ^a^	99.5:0.5	
0.66 ± 0.04 ^a,b^	0.47 ± 0.02 ^a,b^	0.70 ± 0.07 ^a–d^	0.20 ± 0.01 ^a^	99.99:0.01	Water:[Emim][Phe]
0.89 ± 0.05 ^b–f^	0.65 ± 0.01 ^a–d^	0.96 ± 0.03 ^f,g^	0.76 ± 0.05 ^b^	99.9:0.1	
1.00 ± 0.07 ^d–g^	0.74 ± 0.01 ^d–g^	0.99 ± 0.04 ^g^	1.03 ± 0.07 ^c,d^	99.8:0.2	
1.09 ± 0.04 ^f–h^	0.99 ± 0.03 ^h^	1.21 ± 0.06 ^h,i^	1.33 ± 0.07 ^e^	99.7:0.3	
1.80 ± 0.18 ^k,l^	1.44 ± 0.04 ^i^	1.95 ± 0.05 ^o,p^	1.58 ± 0.04 ^e–g^	99.5:0.5	
0.65 ± 0.08 ^a,b^	0.50 ± 0.05 ^a,b^	0.70 ± 0.07 ^a–d^	0.25 ± 0.01 ^a^	99.99:0.01	Water:[Emim][Gly]
1.03 ± 0.06 ^e–h^	0.94 ± 0.05 ^g,h^	0.92 ± 0.02 ^e–g^	0.96 ± 0.14 ^b–d^	99.9:0.1	
1.62 ± 0.05 ^j,k^	1.08 ± 0.09 ^h^	1.43 ± 0.13 ^k,l^	1.52 ± 0.16 ^e,f^	99.8:0.2	
2.02 ± 0.08 ^l–n^	1.77 ± 0.11 ^j^	1.60 ± 0.09 ^l,m^	2.61 ± 0.01 ^i,j^	99.7:0.3	
2.65 ± 0.21 ^p^	2.12 ± 0.11 ^l^	2.11 ± 0.03 ^p^	2.82 ± 0.19 ^j^	99.5:0.5	
0.63 ± 0.03 ^a,b^	0.45 ± 0.02 ^a^	0.69 ± 0.02 ^a–c^	0.19 ± 0.01 ^a^	99.99:0.01	Water:[Bmim][Br]
0.63 ± 0.02 ^a,b^	0.52 ± 0.01 ^a–c^	0.66 ± 0.03 ^a–c^	0.20 ± 0.01 ^a^	99.9:0.1	
0.64 ± 0.02 ^a,b^	0.55 ± 0.01 ^a–d^	0.67 ± 0.03 ^a–c^	0.21 ± 0.01 ^a^	99.8:0.2	
0.65 ± 0.03 ^a,b^	0.56 ± 0.01 ^a–d^	0.68 ± 0.03 ^a–c^	0.21 ± 0.01 ^a^	99.7:0.3	
0.67 ± 0.01 ^a,b^	0.58 ± 0.02 ^a–d^	0.69 ± 0.04 ^a–c^	0.21 ± 0.01 ^a^	99.5:0.5	
0.66 ± 0.04 ^a,b^	0.50 ± 0.01 ^a,b^	0.73 ± 0.04 ^b–d^	0.21 ± 0.01 ^a^	99.99:0.01	Water:[Bmim][Phe]
0.81 ± 0.01 ^a–e^	0.67 ± 0.02 ^b–e^	0.99 ± 0.01 ^g^	0.78 ± 0.06 ^b^	99.9:0.1	
1.02 ± 0.01 ^e–h^	0.72 ± 0.05 ^c–f^	1.00 ± 0.01 ^g^	1.07 ± 0.01 ^d^	99.8:0.2	
1.10 ± 0.01 ^f–h^	0.89 ± 0.01 ^f–h^	1.21 ± 0.02 ^h^	1.46 ± 0.03 ^e^	99.7:0.3	
1.81 ± 0.02 ^k,l^	1.02 ± 0.01 ^h^	1.40 ± 0.14 ^j,k^	1.78 ± 0.13 ^g,h^	99.5:0.5	
0.75 ± 0.08 ^a–d^	0.52 ± 0.02 ^a–c^	0.79 ± 0.06 ^b–f^	0.28 ± 0.04 ^a^	99.99:0.01	Water:[Bmim][Gly]
1.27 ± 0.09 ^h,i^	0.95 ± 0.04 ^h^	1.22 ± 0.01 ^h–j^	0.96 ± 0.11 ^b–d^	99.9:0.1	
1.95 ± 0.10 ^l,m^	1.50 ± 0.34 ^i^	1.75 ± 0.11 ^m,n^	1.85 ± 0.20 ^h^	99.8:0.2	
2.30 ± 0.07 ^o^	2.11 ± 0.15 ^l^	1.97 ± 0.03 ^o,p^	3.24 ± 0.18 ^k^	99.7:0.3	
3.57 ± 0.07 ^q^	2.52 ± 0.06 ^m^	2.30 ± 0.07 ^q^	4.67 ± 0.35 ^l^	99.5:0.5	

Different letters (a–q) are indicative of statistically significant differences within each active compound (one-way ANOVA, Tukey’s test, *p* < 0.05).

**Table 3:** Qualitative and quantitative composition (%, *w*/*w*) for O/W emulsions with and without each IL and/or drug.

	O/W Emulsions			
Composition	Without IL and Drug	With IL and without Drug	With Drug and without IL	With Drug and IL
Crodafos^®^ CES	3.5	3.5	3.5	3.5
Isopropyl myristate	2.0	2.0	2.0	2.0
BHT	0.1	0.1	0.1	0.1
EDTA Na_2_	0.1	0.1	0.1	0.1
Propylene glycol	5.0	5.0	5.0	5.0
Polyethylene glycol 400	5.0	5.0	5.0	5.0
Parabens solution	1.0	1.0	1.0	1.0
Drug	–	-	Maximum solubility ^1^	Maximum solubility ^1^
IL	–	0.2 or 0.5	-	0.2 or 0.5
Triethanolamine	q.s. to pH = 5			
Deionized water	q.s. to 100			

^1^ According to the results obtained in the solubility studies.

**Table 4:** Results from the stability studies of the O/W emulsions prepared in the presence and absence of 0.2% (*v*/*v*) [Cho][Br] or of each of the ILs. Viscosity values were measured after formulation and following six temperature cycles (at −5 °C and 45 °C).

IL	% IL	After Formulation		Stability Studies		
		Visual Analysis	Viscosity (mPas)	After Centrifugation	After Gradual Heating	Viscosity (mPas) after 6 Temperature Cycles
Control 1	-	Stable	5170 ± 90	Unstable	Unstable	-
[Cho][Br]	0.2	Stable	9000 ± 100	Stable	Stable	10,320 ± 80
[Cho][Phe]	0.2	Stable	12,700 ± 102			15,400 ± 100
[Cho][Gly]	0.2	Stable	11,800 ± 52			13,100 ± 105
[Emim][Br]	0.2	Stable	9100 ± 97			11,000 ± 101
[Emim][Phe]	0.2	Stable	10,000 ± 132			12,400 ± 129
[Emim][Gly]	0.2	Stable	10,400 ± 188			13,100 ± 77
[Bmim][Br]	0.2	Stable	8750 ± 65			9500 ± 90
[Bmim][Phe]	0.2	Stable	9100 ± 80			11,000 ± 85
[Bmim][Gly]	0.2	Stable	9200 ± 120			11,400 ± 112

**Table 5:** Results from the stability studies of the O/W emulsions prepared in the presence of each drug individually and in the presence and absence of the glycinate derived ILs. Viscosity values were measured after formulation and following six temperature cycles (at −5 °C and 45 °C).

Drug	IL	% IL	After Formulation		Stability Studies		
			Visual Analysis	Viscosity (mPas)	After Centrifuge	After Gradual Heating	Viscosity (mPas) after 6 Temperature Cycles
Ferulic Acid	Control 2a	-	Stable	8000 ± 80	Unstable	Unstable	-
	[Cho][Gly]	0.2	Stable	12,000 ± 75	Stable	Stable	12,500 ± 100
		0.5	Stable	13,700 ± 110			15,000 ± 90
	[Emim][Gly]	0.2	Stable	11,300 ± 100			12,000 ± 100
	[Bmim][Gly]	0.2	Stable	10,000 ± 130			12,600 ± 100
Caffeic Acid	Control 2b	-	Stable	8500 ± 100	Unstable	Unstable	-
	[Cho][Gly]	0.2	Stable	11,000 ± 95	Stable	Stable	12,000 ± 90
		0.5	Stable	12,000 ± 100			15,500 ± 95
	[Emim][Gly]	0.2	Stable	11,200 ± 90			14,100 ± 80
	[Bmim][Gly]	0.2	Stable	11,000 ± 80			14,500 ± 90
*p*-Coumaric Acid	Control 2c	-	Stable	8200 ± 100	Unstable	Unstable	-
	[Cho][Gly]	0.2	Stable	12,000 ± 100	Stable	Stable	15,500 ± 100
		0.5	Stable	13,500 ± 100			17,000 ± 90
	[Emim][Gly]	0.2	Stable	10,300 ± 90			14,600 ± 100
	[Bmim][Gly]	0.2	Stable	10,200 ± 100			14,000 ± 100
Rutin	Control 2d	-	Stable	7500 ± 150	Unstable	Unstable	-
	[Cho][Gly]	0.2	Stable	12,800 ± 100	Stable	Stable	13,100 ± 105
		0.5	Stable	13,400 ± 90			16,000 ± 100
	[Emim][Gly]	0.2	Stable	10,400 ± 188			13,100 ± 77
	[Bmim][Gly]	0.2	Stable	9220 ± 50			11,140 ± 52

**Table 6:** Results from the accelerated and shelf-life stability studies of the O/W emulsions prepared in the presence of 0.2% (*v*/*v*) of [Cho][Br] or of each of the ILs and without the drug. Viscosity values were measured after 90 days in an oven (40 ± 2 °C), in a refrigerator (5 ± 2 °C), or at room temperature.

IL	% IL	Accelerated Stability		Shelf Test
		Heating at Oven	Cooling at Refrigerator	Viscosity (mPas)
[Cho][Br]	0.2	12,100 ± 100	12215 ± 120	13,400 ± 50
[Cho][Phe]	0.2	15,500 ± 85	14950 ± 80	16,200 ± 65
[Cho][Gly]	0.2	15,220 ± 50	15000 ± 80	16,050 ± 100
[Emim][Br]	0.2	11,900 ± 100	11500 ± 50	13,100 ± 90
[Emim][Phe]	0.2	12,200 ± 100	11950 ± 50	13,450 ± 70
[Emim][Gly]	0.2	12,450 ± 100	12320 ± 100	14,120 ± 90
[Bmim][Br]	0.2	9500 ± 100	9900 ± 100	9900 ± 50
[Bmim][Phe]	0.2	10,300 ± 50	10220 ± 100	11,000 ± 100
[Bmim][Gly]	0.2	10,175 ± 50	10300 ± 70	11,110 ± 50

**Table 7:** Results from the accelerated and shelf-life stability studies of the O/W emulsions prepared in the presence of each drug individually and in the presence of the glycinate derived ILs. Viscosity values were measured after 90 days in an oven (40 ± 2 °C), in a refrigerator (5 ± 2 °C), or at room temperature.

Drug	IL	% IL	Accelerated Stability		Shelf Test
			Heating at Oven	Cooling at Refrigerator	Viscosity (mPas)
Ferulic Acid	[Cho][Gly]	0.2	15,500 ± 100	15,410 ± 50	16,250 ± 100
		0.5	16,300 ± 50	16,570 ± 110	17,120 ± 120
	[Emim][Gly]	0.2	12,320 ± 80	12,200 ± 50	14,200 ± 110
	[Bmim][Gly]	0.2	11,000 ± 50	10,900 ± 50	12,100 ± 100
Caffeic Acid	[Cho][Gly]	0.2	13,100 ± 50	13,310 ± 80	14,850 ± 50
		0.5	13,300 ± 60	13,140 ± 100	15,680 ± 50
	[Emim][Gly]	0.2	12,520 ± 100	12,600 ± 100	14,200 ± 150
	[Bmim][Gly]	0.2	12,100 ± 100	11,990 ± 100	14,620 ± 80
*p*-Coumaric Acid	[Cho][Gly]	0.2	13,250 ± 50	13,500 ± 100	16,000 ± 100
		0.5	13,900 ± 50	14,540 ± 100	17,120 ± 150
	[Emim][Gly]	0.2	11,950 ± 50	12,000 ± 100	14,850 ± 100
	[Bmim][Gly]	0.2	11,400 ± 50	11,355 ± 100	14,700 ± 100
Rutin	[Cho][Gly]	0.2	15,800 ± 50	16,000 ± 100	16,550 ± 120
		0.5	16,750 ± 60	17,010 ± 100	18,225 ± 115
	[Emim][Gly]	0.2	12,450 ± 50	12,800 ± 100	14,780 ± 100
	[Bmim][Gly]	0.2	11,500 ± 50	11,650 ± 100	12,380 ± 100

## Supplementary Materials

Supplementary Materials can be found at https://www.mdpi.com/article/10.3390/ijms22094338/s1.
