# Supplementary material for: Upgrading the Topical Delivery of Poorly Soluble Drugs Using Ionic Liquids as a Versatile Tool
Source: Int J Mol Sci. 2021 Apr 21;22(9):4338. doi: 10.3390/ijms22094338 (PMC8122351; doi:10.3390/ijms22094338)
Supplement: Supplementary file 1 [file ijms-22-04338-s001.zip › ijms-1167731-supplementary.pdf]

SM1: ¹H NMR (400 MHz), ¹³C NMR (100.4 MHz) and FTIR spectra of the eight prepared ionic liquids, [Cho][Phe] (**1**), [Cho][Gly] (**2**), [Emim][Br] (**3**), [Emim][Phe] (**4**), [Emim][Gly] (**5**), [Bmim][Br] (**6**), [Bmim][Phe] (**7**), [Bmim][Gly] (**8**).

(1) [Cho][Phe]

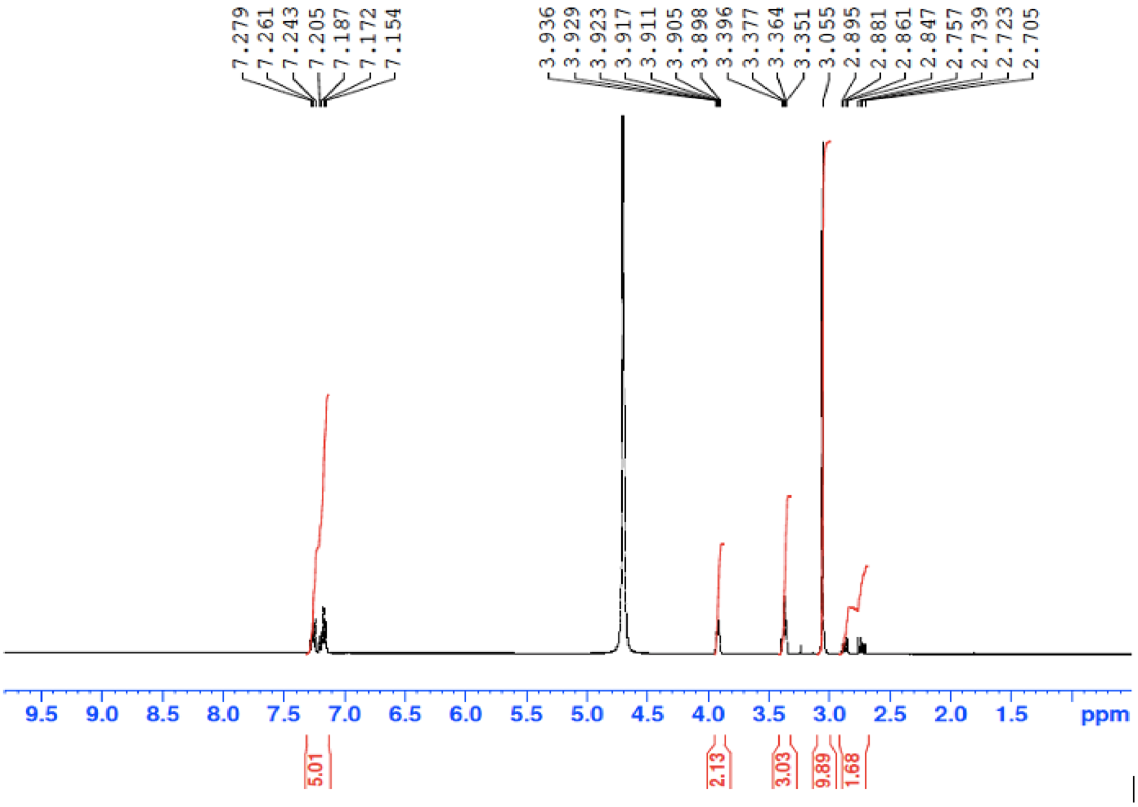

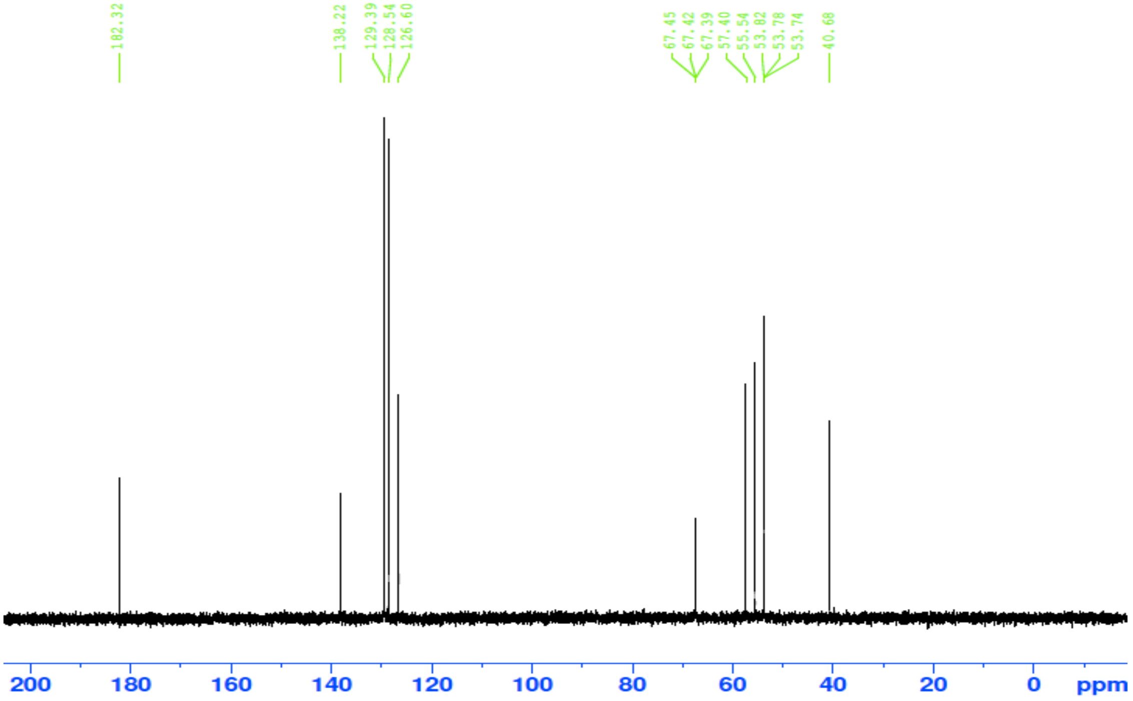

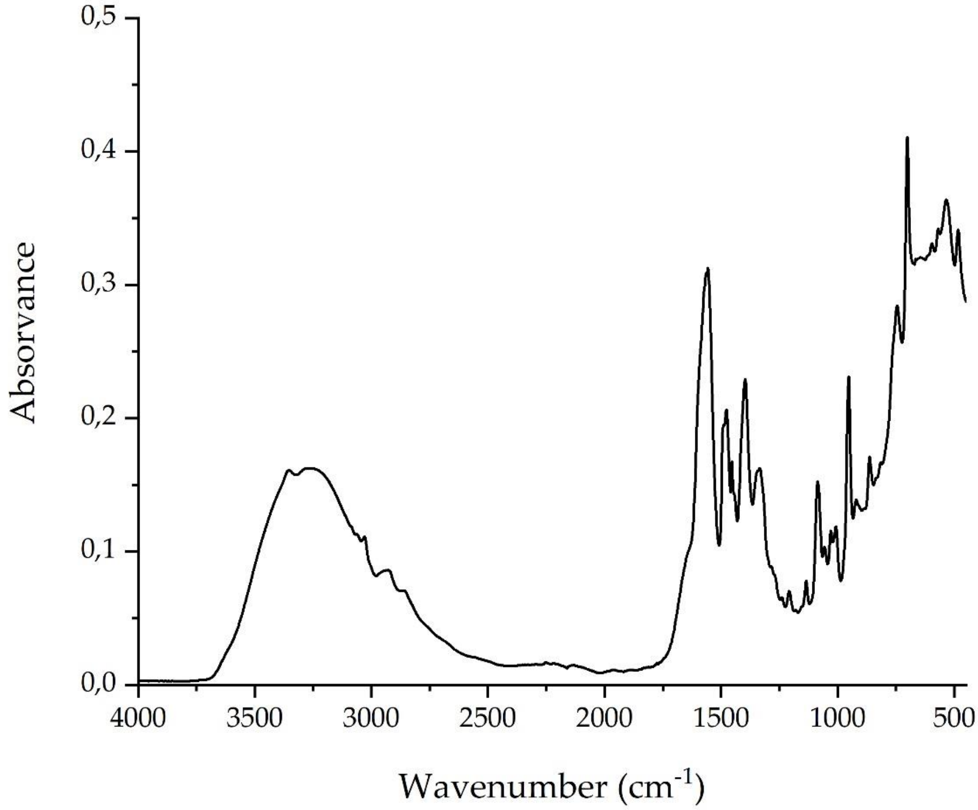
Absorbance
0,0
0,1
0,2
0,3
0,4
0,5
4000
3500
3000
2500
2000
1500
1000
500
Wavenumber (cm^{-1})

(2) [Cho][Gly]

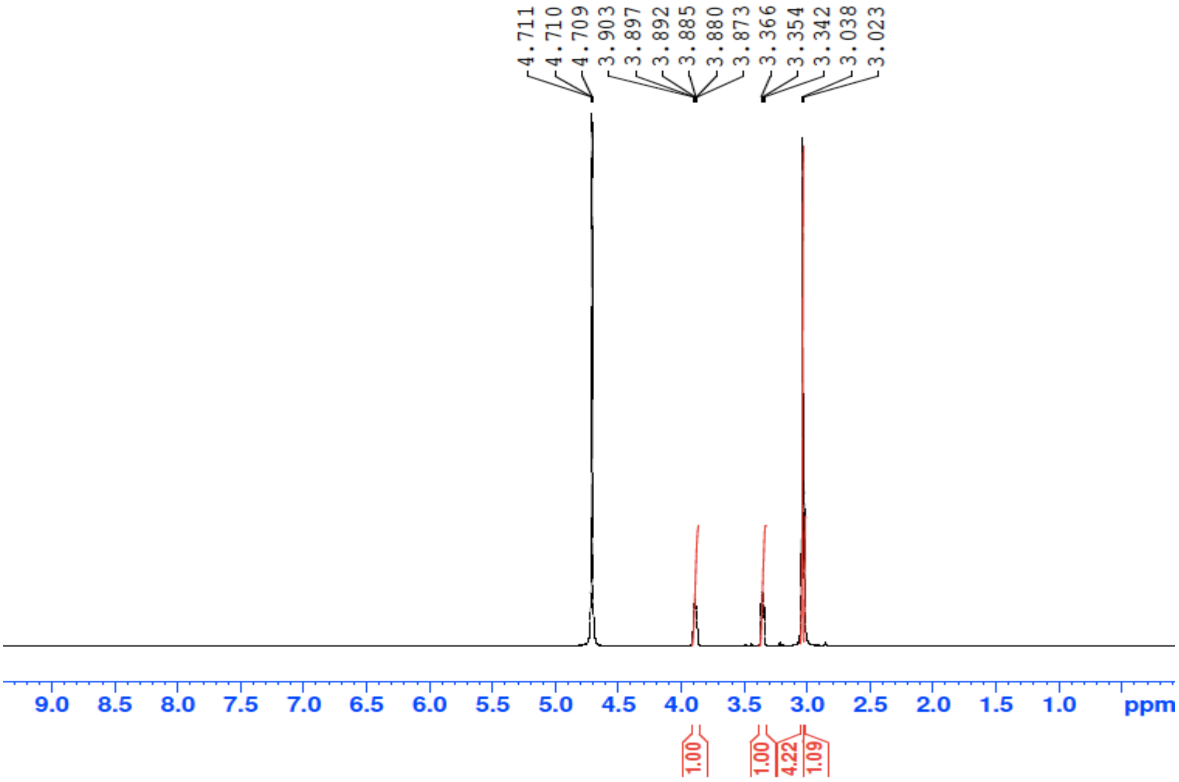
4.711
4.710
4.709
3.903
3.897
3.892
3.885
3.880
3.873
3.366
3.354
3.342
3.038
3.023
9.0 8.5 8.0 7.5 7.0 6.5 6.0 5.5 5.0 4.5 4.0 3.5 3.0 2.5 2.0 1.5 1.0 ppm
1.00
1.00
4.22
1.09

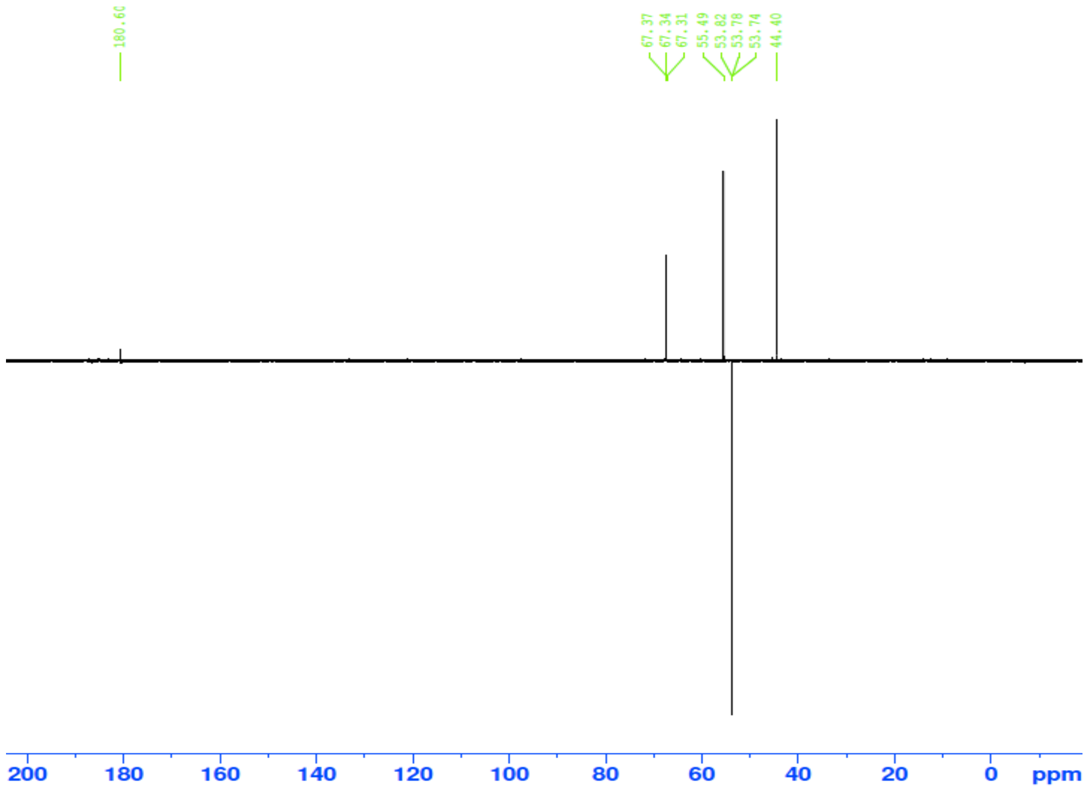
180.60
67.37
67.34
67.31
55.49
53.82
53.78
53.74
44.40
200
180
160
140
120
100
80
60
40
20
0
ppm

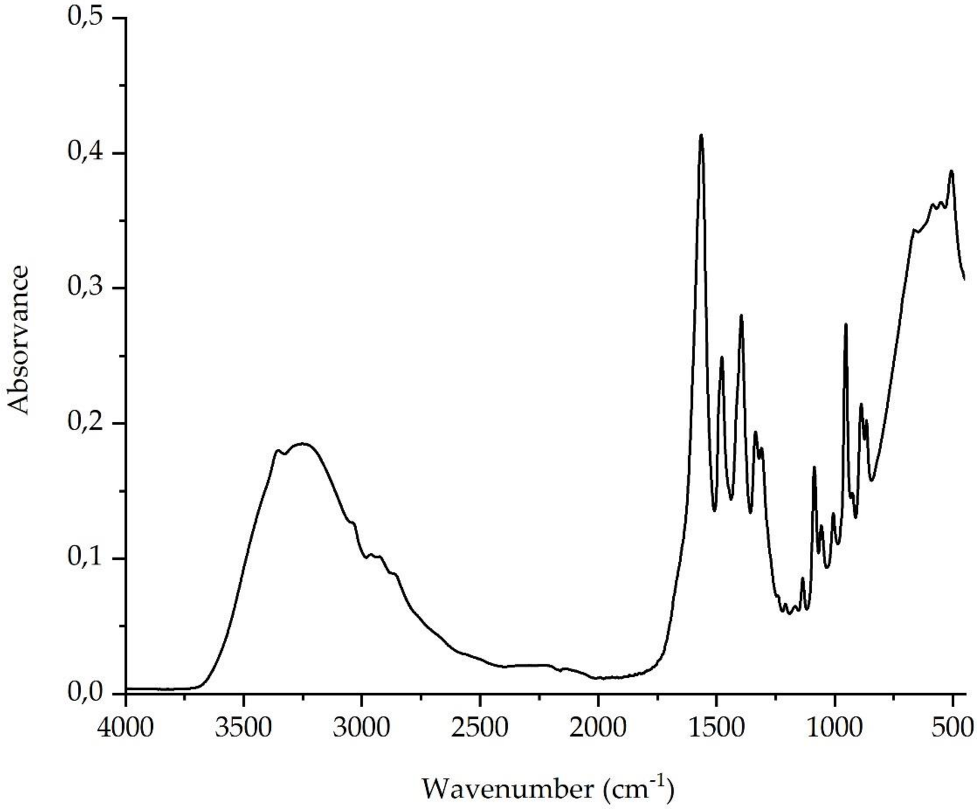
Absorvance
0,5
0,4
0,3
0,2
0,1
0,0
4000
3500
3000
2500
2000
1500
1000
500
Wavenumber (cm-1)

(3) [Emim][Br]

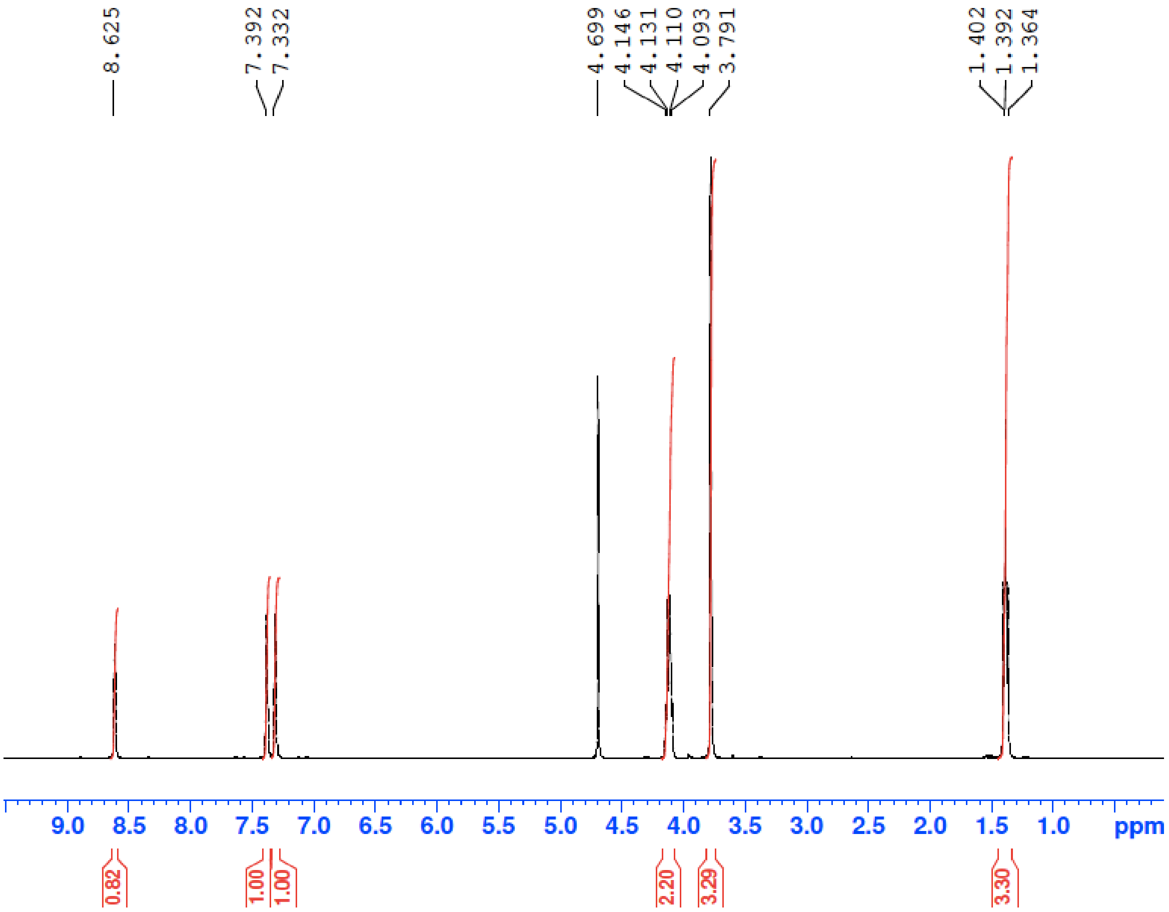
8.625
7.392
7.332
4.699
4.146
4.131
4.110
4.093
3.791
1.402
1.392
1.364
9.0
8.5
8.0
7.5
7.0
6.5
6.0
5.5
5.0
4.5
4.0
3.5
3.0
2.5
2.0
1.5
1.0
ppm
0.82
1.00
1.00
2.20
3.29
3.30

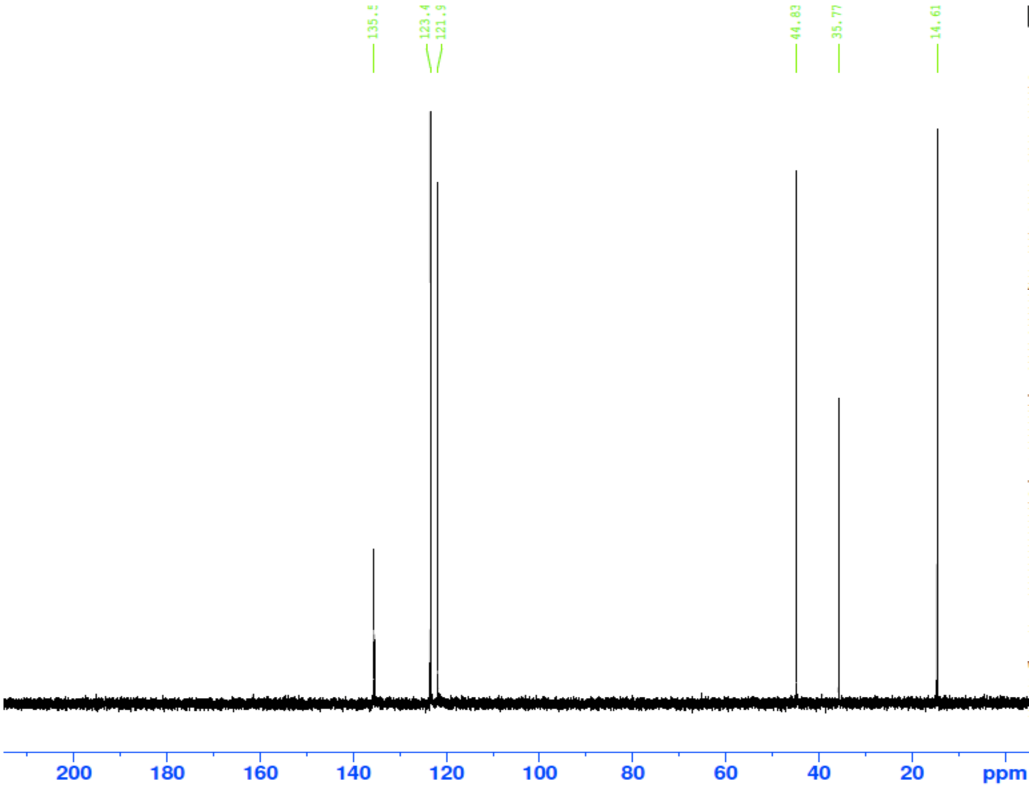
123.4
121.9
44.83
35.77
14.61
200
180
160
140
120
100
80
60
40
20
ppm

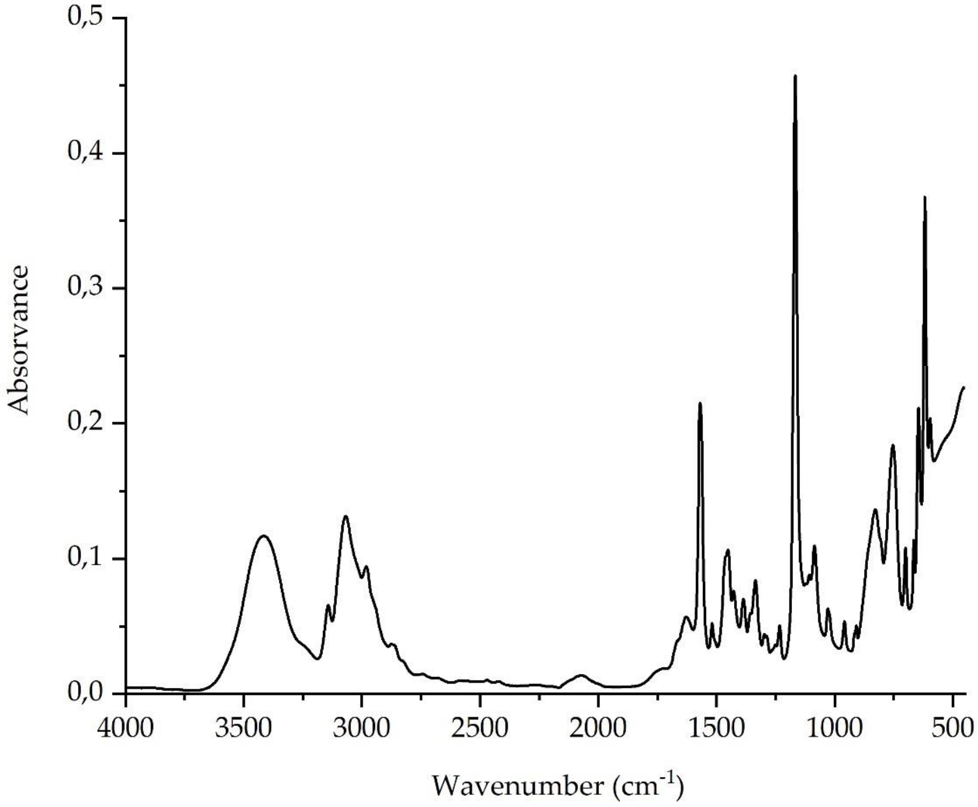

0,5
0,4
0,3
0,2
0,1
0,0
Absorvance
4000
3500
3000
2500
2000
1500
1000
500
Wavenumber (cm-1)

(4) [Emim][Phe]

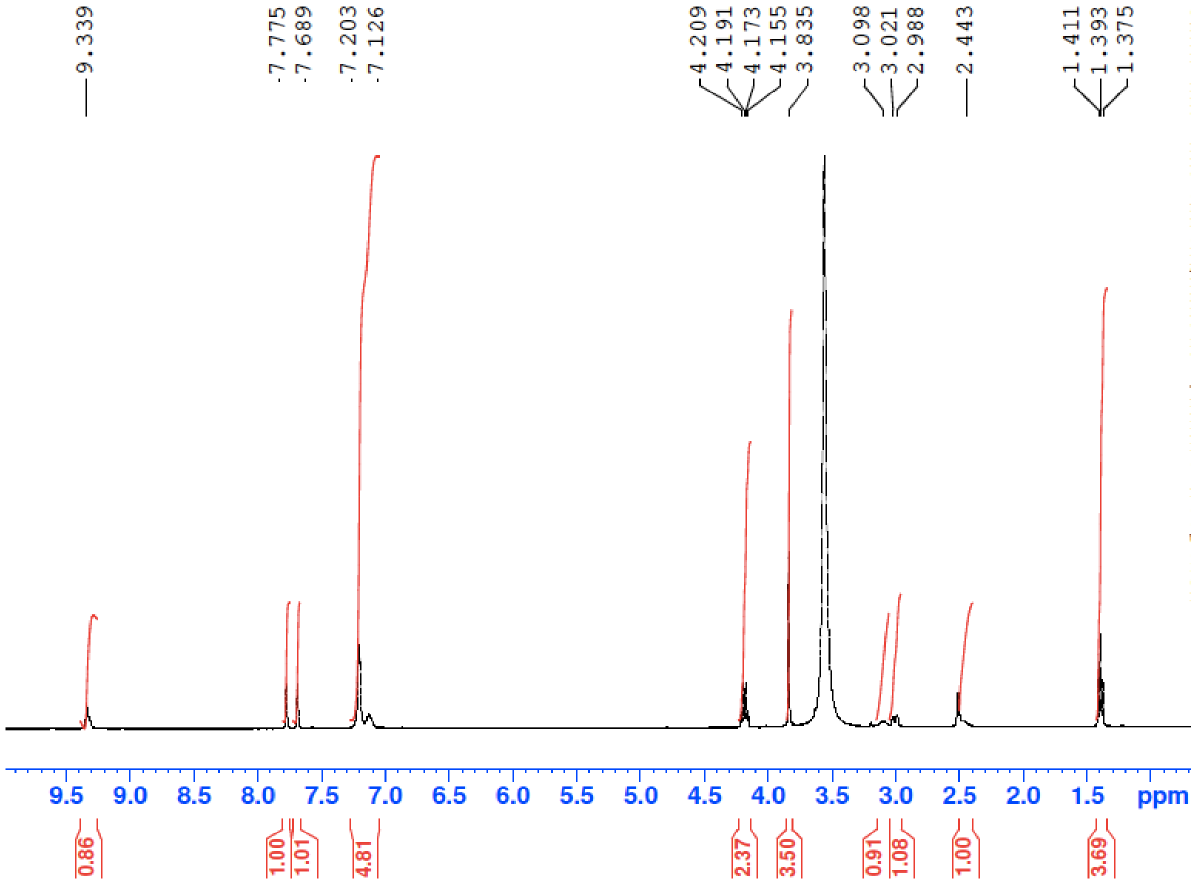
9.339
7.775
7.689
7.203
7.126
4.209
4.191
4.173
4.155
3.835
3.098
3.021
2.988
2.443
1.411
1.393
1.375
9.5 9.0 8.5 8.0 7.5 7.0 6.5 6.0 5.5 5.0 4.5 4.0 3.5 3.0 2.5 2.0 1.5 ppm
0.86
1.00
1.01
4.81
2.37
3.50
0.91
1.08
1.00
3.69

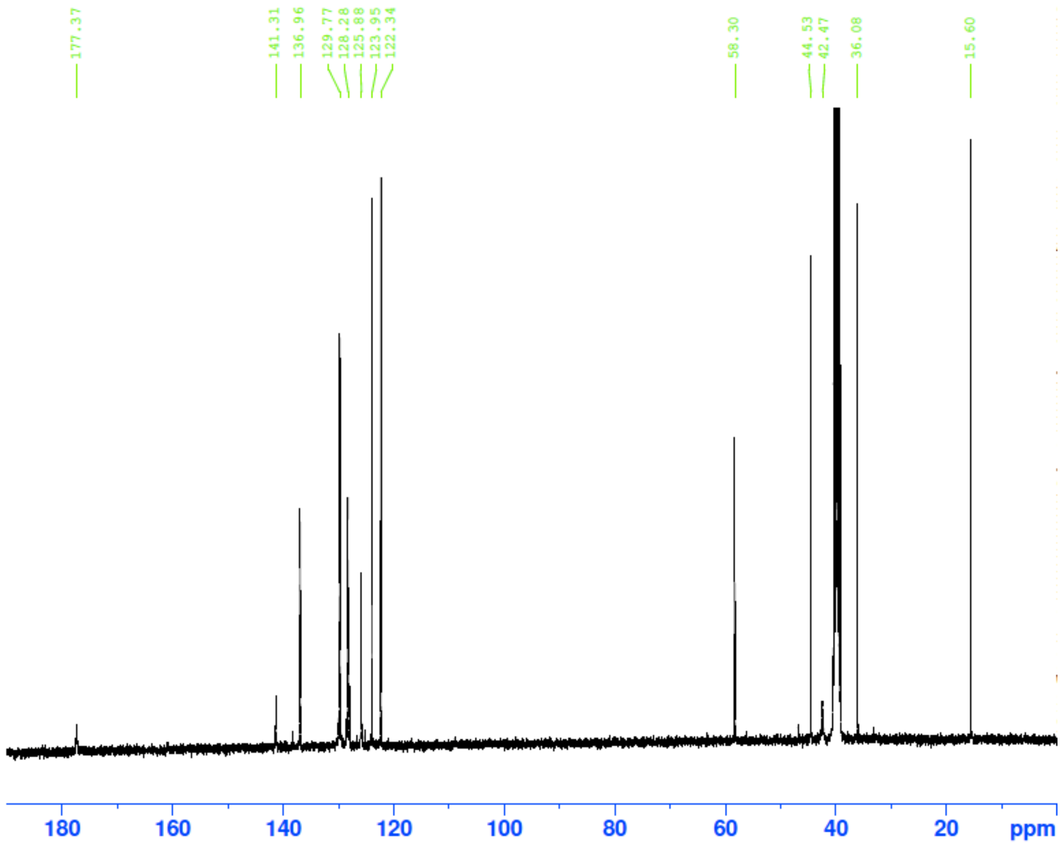
177.37
141.31
136.96
129.77
128.28
125.88
123.95
122.34
58.30
44.53
42.47
36.08
15.60
180 160 140 120 100 80 60 40 20 ppm

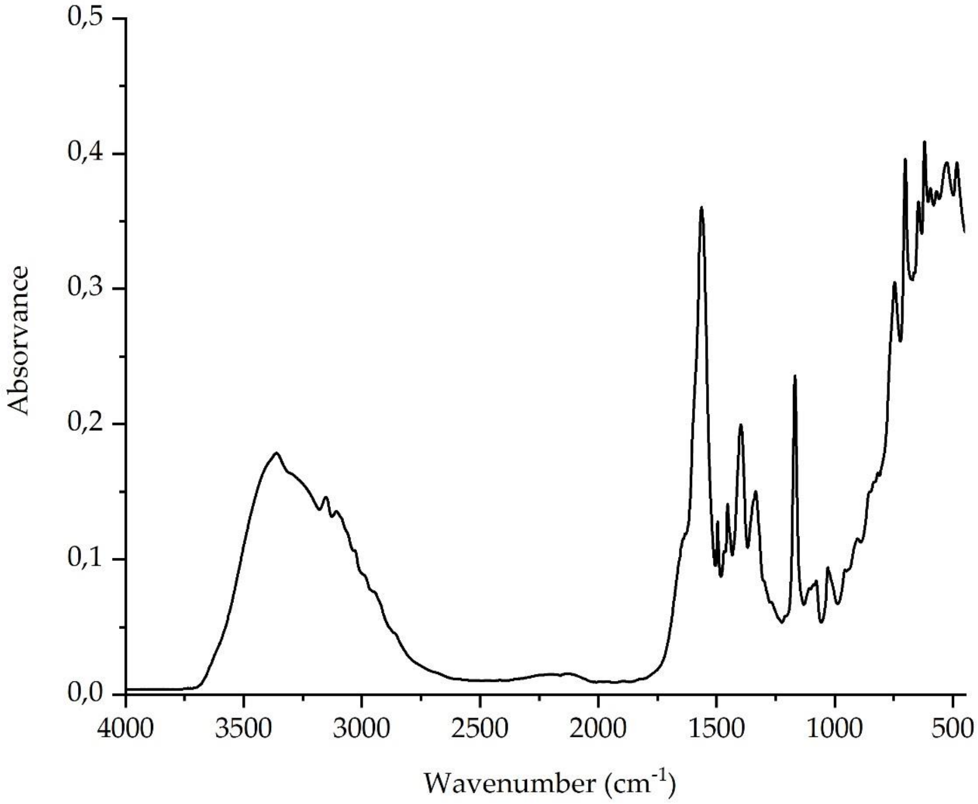

0,5
0,4
0,3
0,2
0,1
0,0
Absorvance
4000
3500
3000
2500
2000
1500
1000
500
Wavenumber (cm-1)

(5) [Emim][Gly]

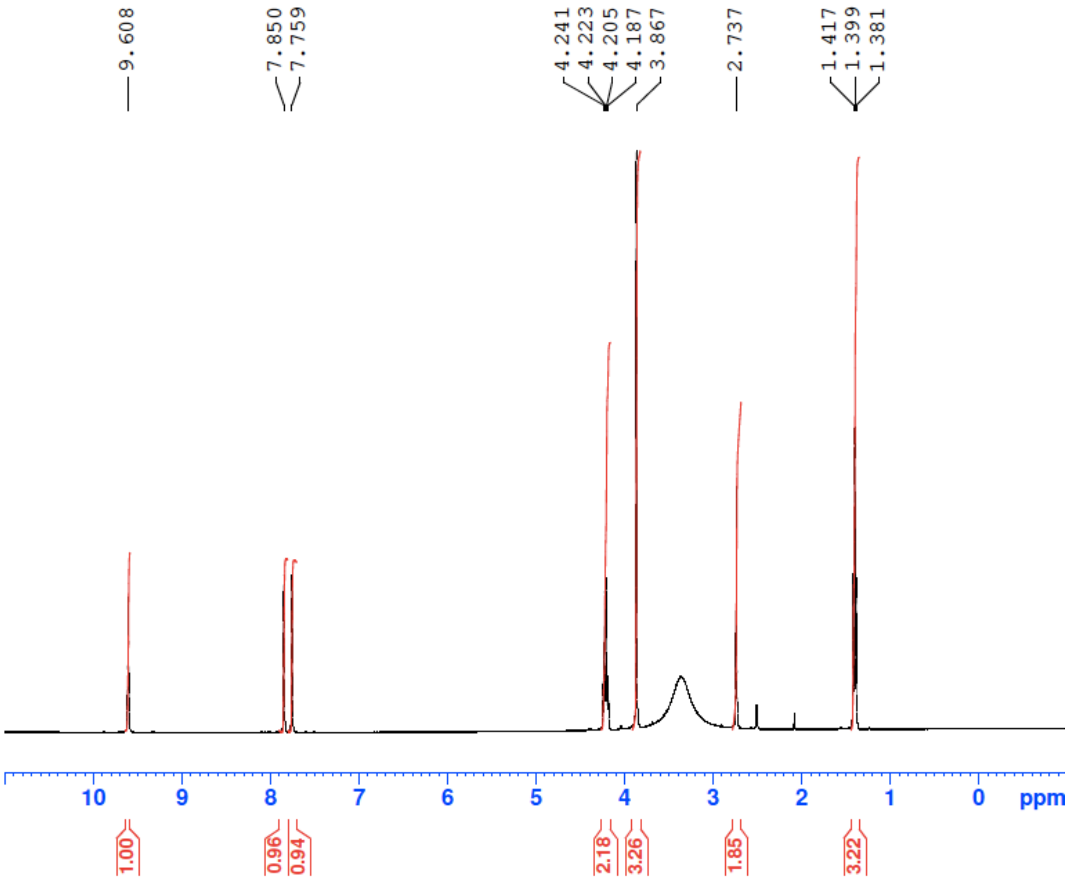
9.608
7.850
7.759
4.241
4.223
4.205
4.187
3.867
2.737
1.417
1.399
1.381
10 9 8 7 6 5 4 3 2 1 0 ppm
1.00
0.96
0.94
2.18
3.26
1.85
3.22

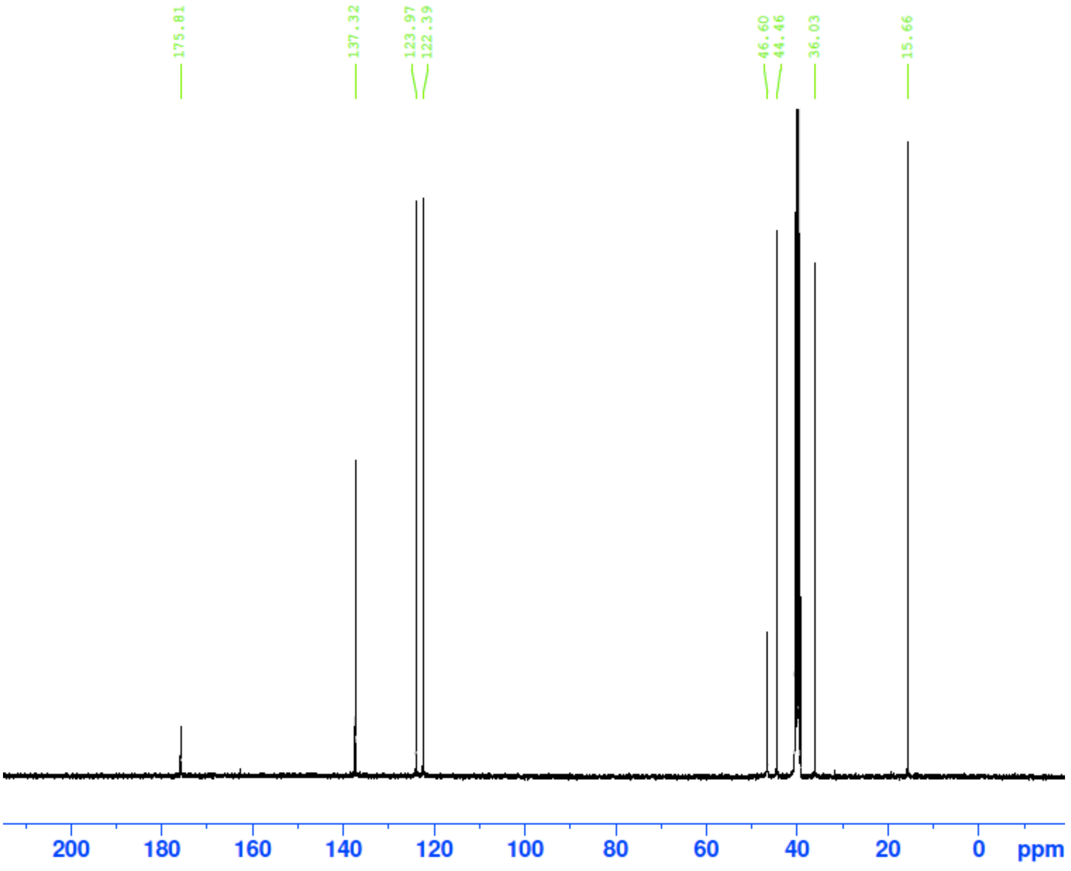
175.81
137.32
123.97
122.39
46.60
44.46
36.03
15.66
200 180 160 140 120 100 80 60 40 20 0 ppm

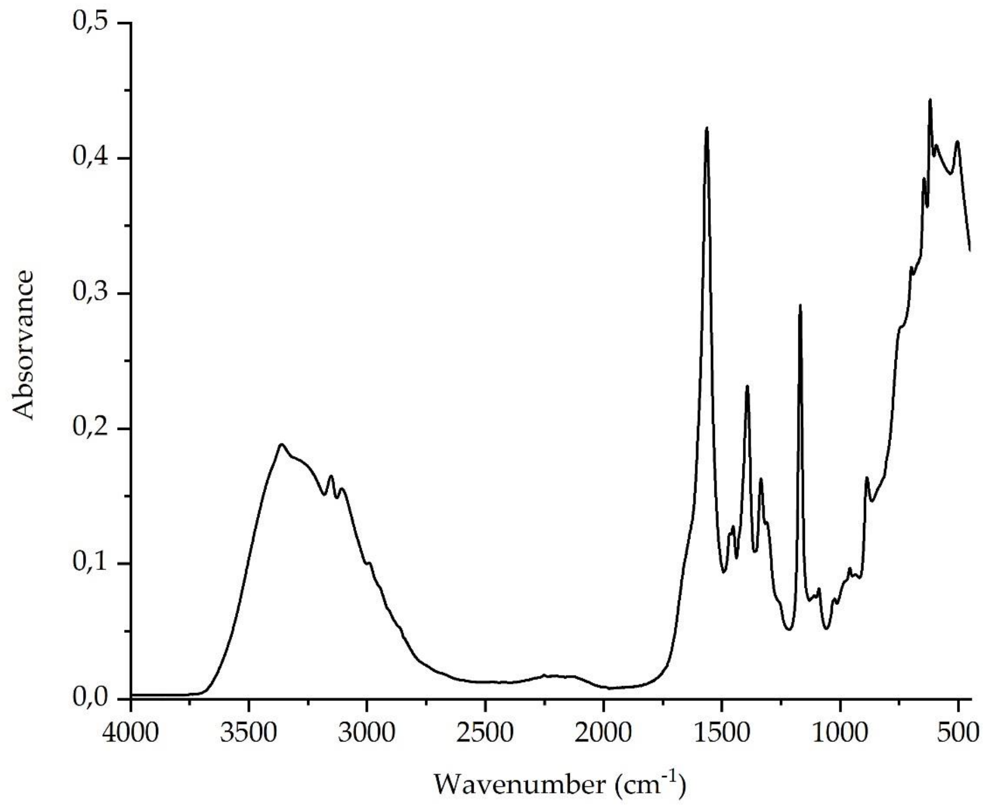

Absorvance
0,0
0,1
0,2
0,3
0,4
0,5
4000
3500
3000
2500
2000
1500
1000
500
Wavenumber (cm-1)

(6) Bmim][Br]

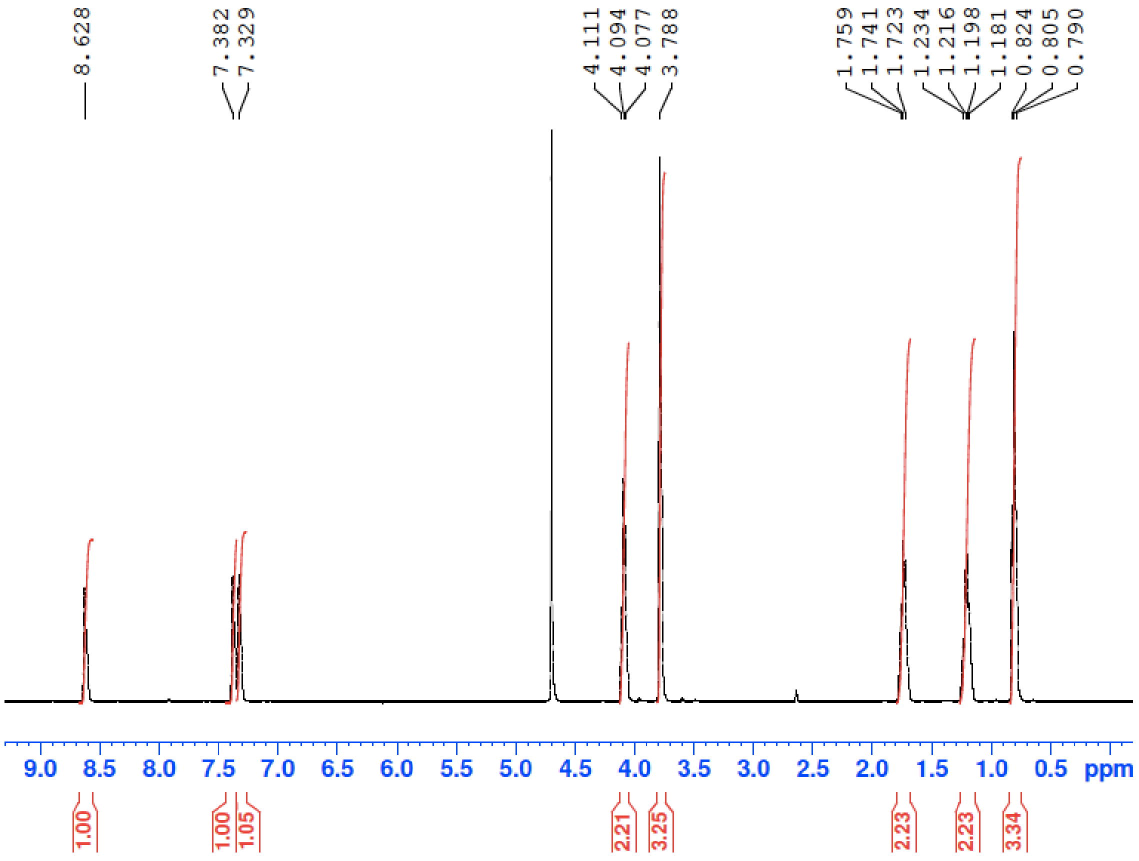

8.628
7.382
7.329
4.111
4.094
4.077
3.788
1.759
1.741
1.723
1.234
1.216
1.198
1.181
0.824
0.805
0.790
9.0 8.5 8.0 7.5 7.0 6.5 6.0 5.5 5.0 4.5 4.0 3.5 3.0 2.5 2.0 1.5 1.0 0.5 ppm
1.00
1.00
1.05
2.21
3.25
2.23
2.23
3.34

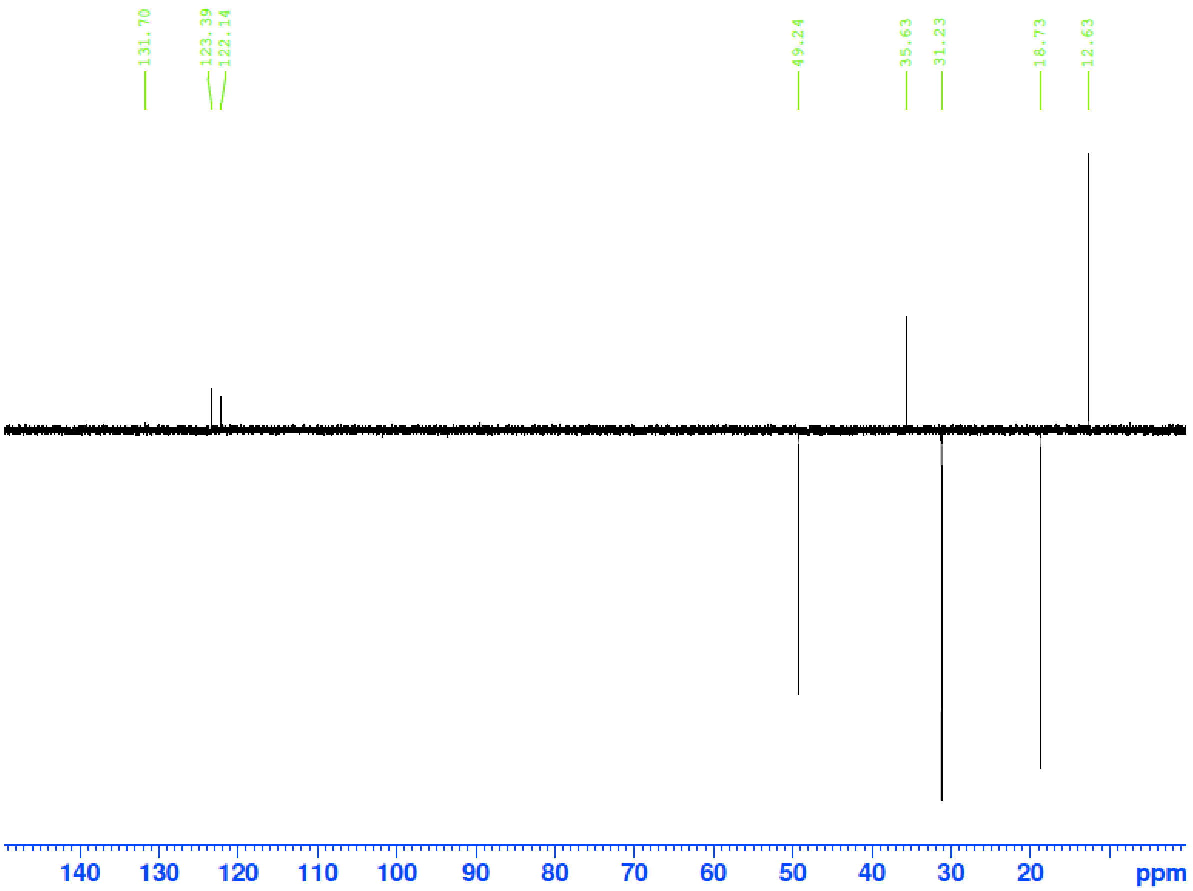
131.70
123.39
122.14
49.24
35.63
31.23
18.73
12.63
140
130
120
110
100
90
80
70
60
50
40
30
20
ppm

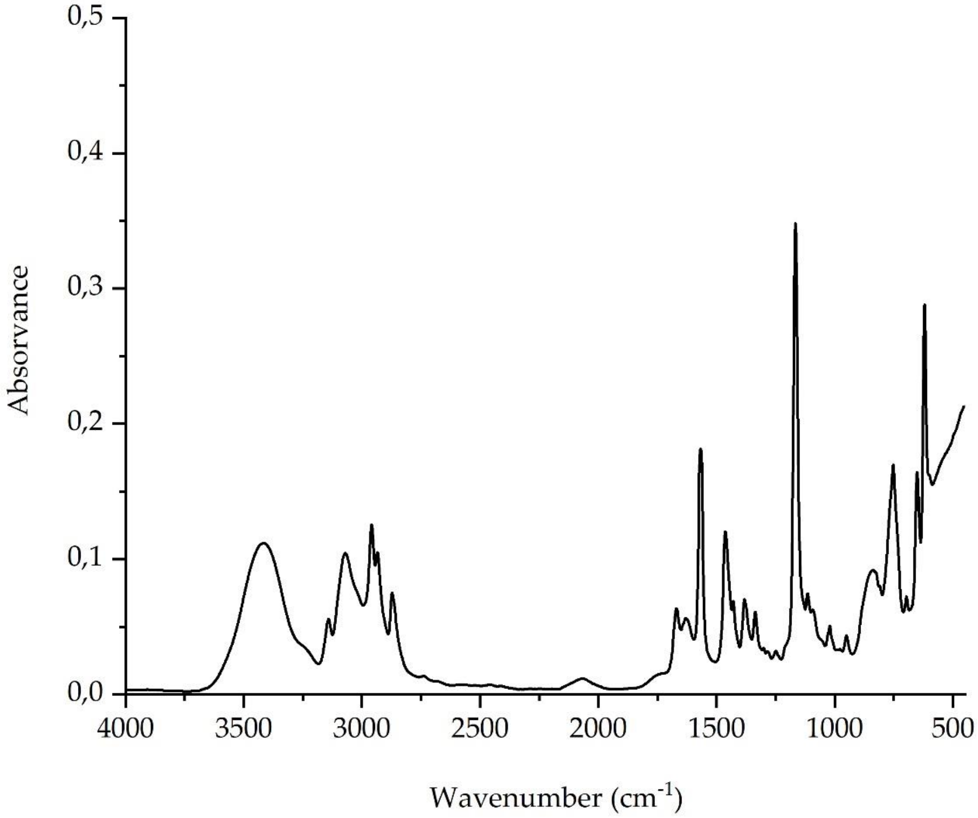
Absorvance
0,0
0,1
0,2
0,3
0,4
0,5
4000
3500
3000
2500
2000
1500
1000
500
Wavenumber (cm-1)

(7) Bmim][Phe]

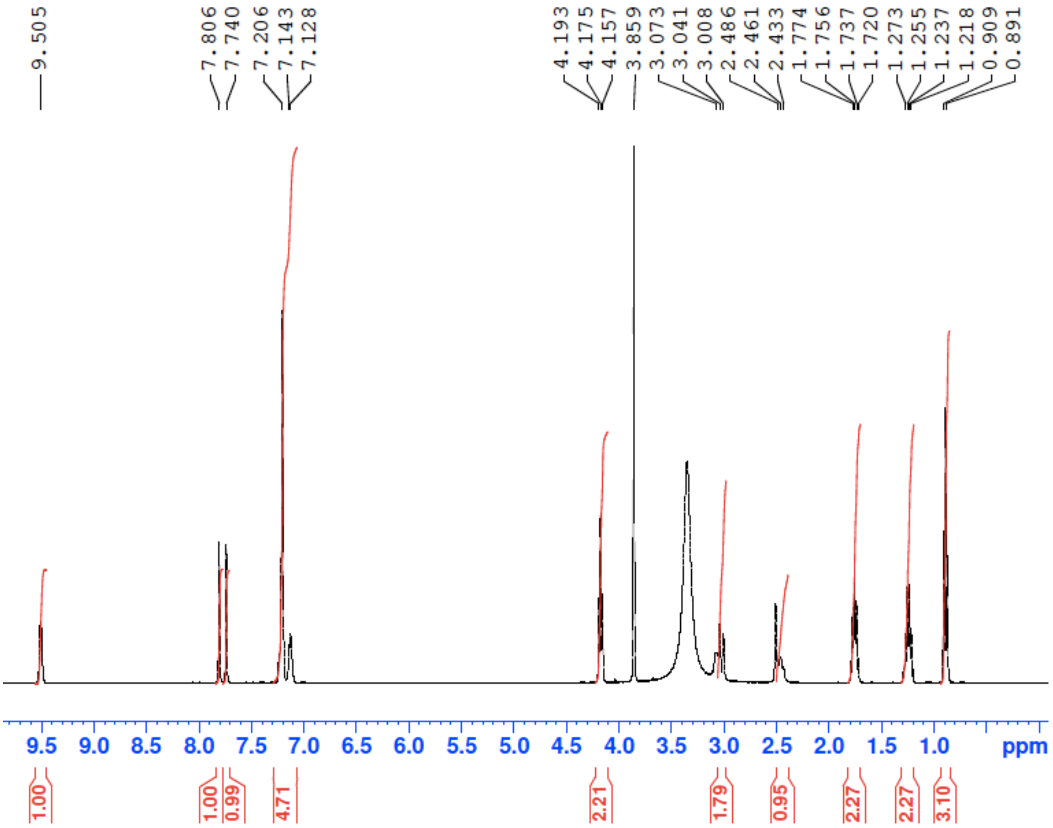
9.505
7.806
7.740
7.206
7.143
7.128
4.193
4.175
4.157
3.859
3.073
3.041
3.008
2.486
2.461
2.433
1.774
1.756
1.737
1.720
1.273
1.255
1.237
1.218
0.909
0.891
9.5 9.0 8.5 8.0 7.5 7.0 6.5 6.0 5.5 5.0 4.5 4.0 3.5 3.0 2.5 2.0 1.5 1.0 ppm
1.00
1.00
0.99
4.71
2.21
1.79
0.95
2.27
2.27
3.10

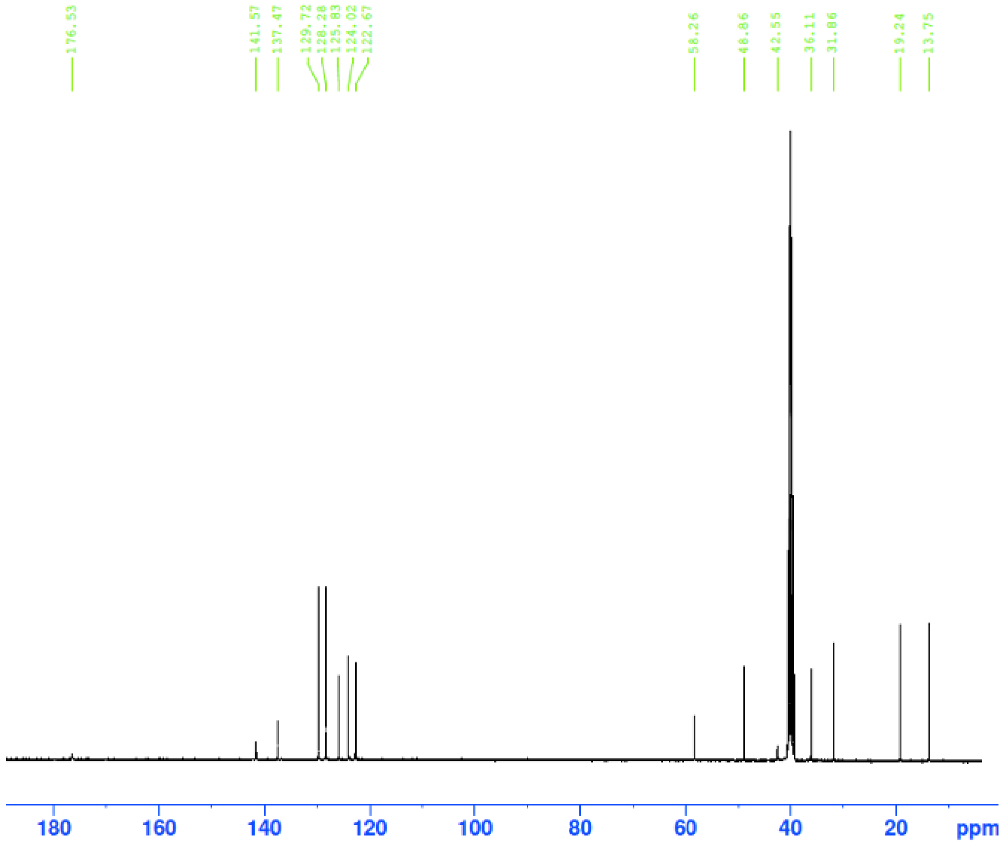
176.53
141.57
137.47
129.72
128.28
125.83
124.02
122.67
58.26
48.86
42.55
36.11
31.86
19.24
13.75
180 160 140 120 100 80 60 40 20 ppm

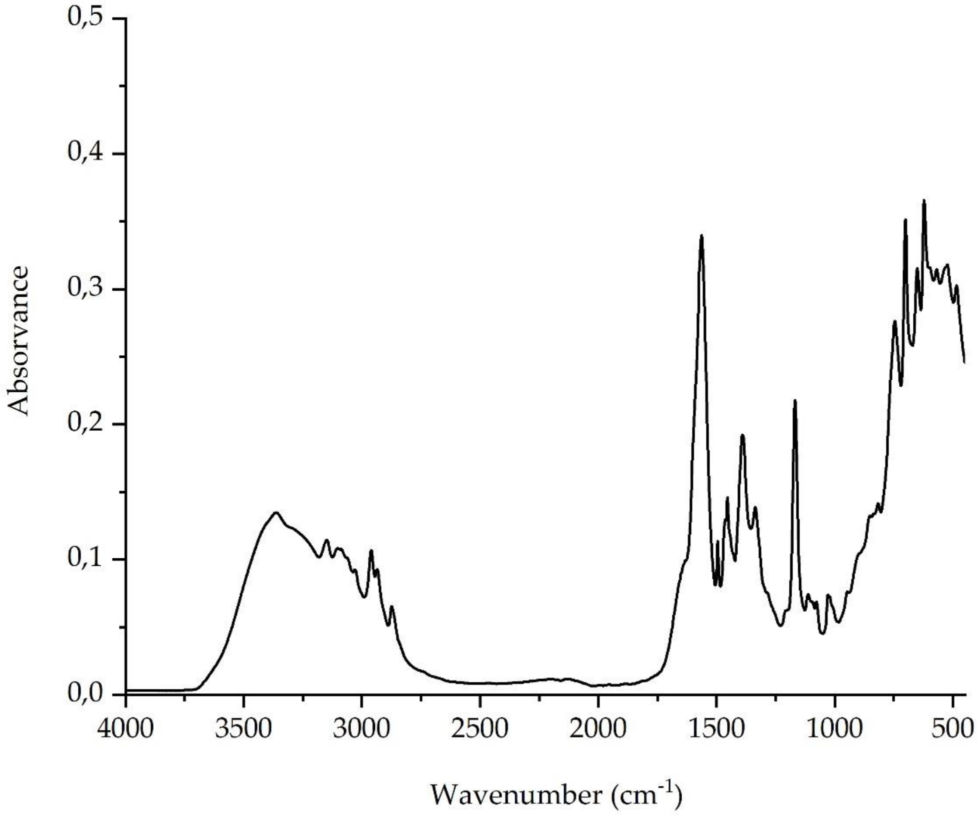

0,5
0,4
0,3
0,2
0,1
0,0
Absorvance
4000
3500
3000
2500
2000
1500
1000
500
Wavenumber (cm-1)

(8) Bmim][Gly]

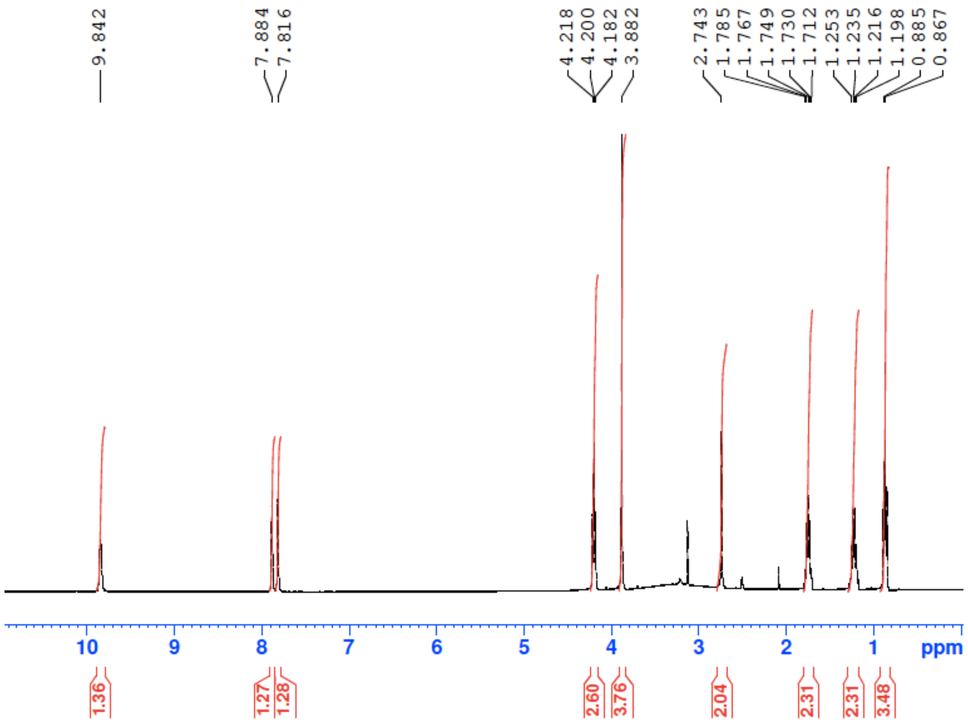

9.842
7.884
7.816
4.218
4.200
4.182
3.882
2.743
1.785
1.767
1.749
1.730
1.712
1.253
1.235
1.216
1.198
0.885
0.867
10
9
8
7
6
5
4
3
2
1
ppm
1.36
1.27
1.28
2.60
3.76
2.04
2.31
2.31
3.48

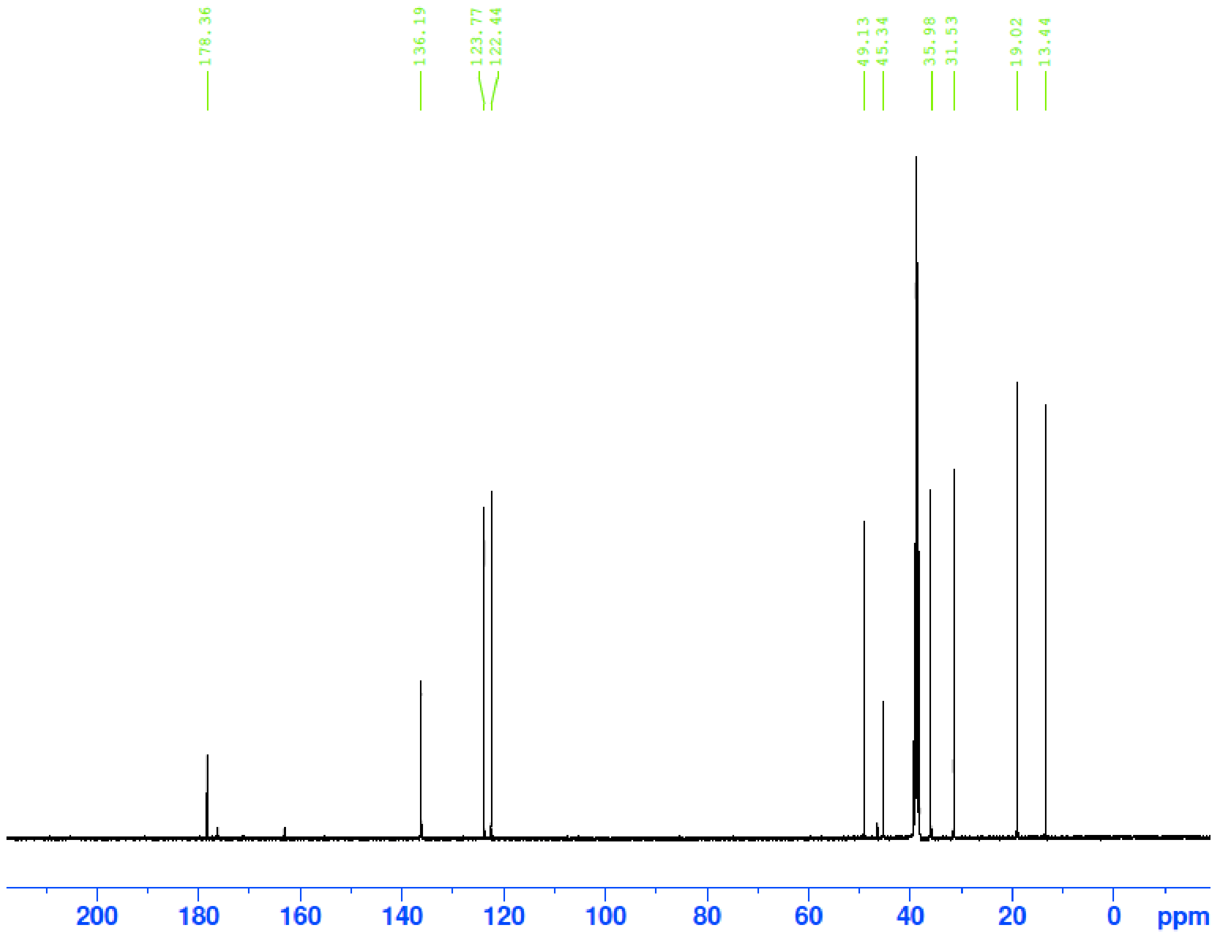

178.36
136.19
123.77
122.44
49.13
45.34
35.98
31.53
19.02
13.44
200
180
160
140
120
100
80
60
40
20
0
ppm

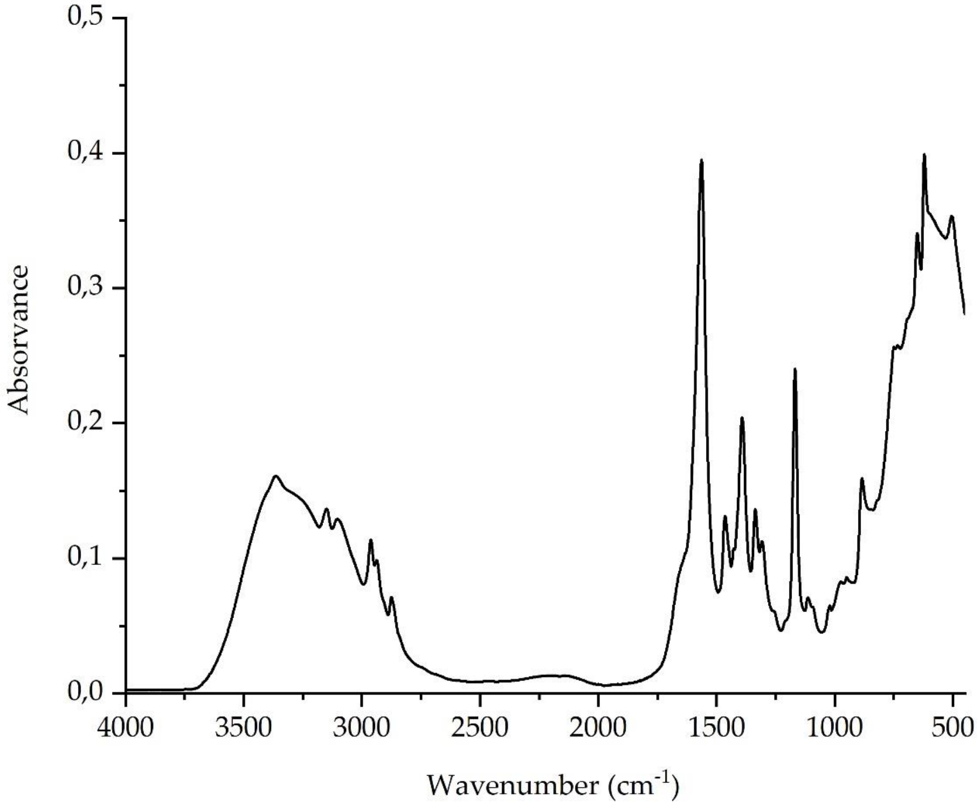

Absorvance
0,0
0,1
0,2
0,3
0,4
0,5
4000
3500
3000
2500
2000
1500
1000
500
Wavenumber (cm-1)
